# A systematic review and meta‐analysis on the relationship of eco‐emotions on the mental health and wellbeing of young adults

**DOI:** 10.1111/aphw.70157

**Published:** 2026-05-06

**Authors:** Samridha S. J. B. Rana, Paul Graham Morris, Caroline E. Brett, Emily Pacheco, Kalliopi Demetriou, Hamdullah Tunç

**Affiliations:** ^1^ School of Health in Social Science, Old Medical School, Elsie Inglis Quadrangle, Teviot Pl University of Edinburgh Edinburgh UK; ^2^ Clinical Psychology NHS Grampian UK; ^3^ Department of Psychological Counselling and Guidance Hacettepe University Ankara Türkiye

**Keywords:** eco‐anger, eco‐anxiety, eco‐emotions, eco‐fear, mental health, young adults

## Abstract

This paper systematically reviews the literature on levels of eco‐emotions reported by young adults (aged 18–29) across nations, with meta‐analyses of associations between eco‐anxiety and mental health outcomes. Fourteen databases were searched for relevant studies. Fifty‐nine studies reported levels of eco‐emotions, with 37 studies reporting associations between eco‐anxiety and each of anxiety, depression, and stress. Eco‐anxiety was the most reported eco‐emotion. Higher scores of eco‐anxiety and eco‐fear were reported by populations facing direct environmental impacts. Eco‐anger and eco‐hope were reported to have a role in adaptive coping. Meta‐analyses indicated moderate, significant positive associations between eco‐anxiety and depression *r* = 0.29, anxiety *r* = 0.34, and stress *r* = 0.30. Meta‐regressions were conducted to explore heterogeneity. Our results highlight the importance of addressing heterogeneity in operationalizing the construct of eco‐anxiety and the need to collect data on eco‐emotions in low‐and‐middle‐income nations, which is lacking in the current literature.

## INTRODUCTION

Human activities have pushed ecosystems to levels previously not experienced globally, leading to anthropogenic environmental degradation accelerating at an alarming, unrestrained pace, as reflected in increases in extreme weather events and natural disasters. The Intergovernmental Panel on Climate Change's (IPCC) sixth assessment report (2023) states “Human activities, principally through emissions of greenhouse gases, have unequivocally caused global warming, with global surface temperature reaching 1.1°C above 1850‐1900 in 2011‐2020” (IPCC, 2023, p. 6).

Air pollution, melting ice sheets, rising sea levels, deforestation, floods, landslides, drought, heatwaves, and forest fires are contributing to food, water, and energy insecurities (Jyoteeshkumar Reddy et al., 2021; Krishnan, 2022; Lawrence et al., 2007; MacDonald et al., 2013; Pietromarchi, 2021), which are being experienced increasingly across the globe. (Cai et al., 2015; Hertel & Lobell, 2014; Hertel & Rosch, 2010; Mbow et al., 2019; Suri, 2022). From economic losses to political upheaval, displacement, migration, physical injury and psychological problems, to trauma and death, the consequences of the climate and ecological crises and extreme weather events are severe, numerous, and increasing (Brown, 2008; Hanigan et al., 2012; IPCC, 2023).

Faced with an existential threat with the potential to lead to the extinction of humans, feelings of dread, anxiety, and fear abound. Recently, the effects of the climate and ecological crises on mental health and well‐being have been the focus of an increasing number of studies. Research has shown that natural disasters have a detrimental effect on the mental health of survivors (Albrecht et al., 2007; Berry, 2009; Berry et al., 2010; Berry et al., 2011; Coyle & Susteren, 2012; Hanigan et al., 2012; Krishnan, 2022; Lawrence et al., 2007). The rise in frequency and intensity of extreme‐weather events will affect ever‐increasing numbers of people, leading to climate and ecological emotions (i.e. eco‐emotions) and associated mental health difficulties. Significant eco‐emotions have been observed among young children and adolescents (Hickman, 2019; Lehtonen & Pihkala, 2021;Ojala, 2012; Parry et al., 2022) and adults (Clayton & Karazsia, 2020; Heeren et al., 2022; Hickman et al., 2021; Hogg et al., 2021; MacDonald et al., 2013; Searle & Gow, 2010; Stanley et al., 2021). Eco‐anxiety, eco‐fear, eco‐depression, eco‐hopelessness, and eco‐guilt have been found to have debilitating effects on behavioral and social aspects, ranging from social and personal behavioral disengagement (Clayton & Karazsia, 2020; Hogg et al., 2021; Leviston et al., 2021; Searle & Gow, 2010; Stanley et al., 2021). Conversely, the activating nature of eco‐anger and eco‐hope may motivate individuals to take action in addressing the crisis by engaging in climate protests and pro‐environmental behaviors, suggesting the role of adaptive responses (Hogg et al., 2021; Ojala, 2012; Stanley et al., 2021; Stanley et al., 2025).

From a theoretical perspective, eco‐emotions may be understood within a stress and coping framework, whereby environmental threats are appraised as exceeding one's coping resources (Lazarus & Folkman, 1984). Appraisal theories further suggest that distinct eco‐emotions such as fear, anger, and hope arise from different cognitive evaluations of these threats (Roseman, 1996; Scherer, 2001). Additionally, the large‐scale and future‐oriented nature of the climate and ecological crises aligns with existential perspectives, which emphasize emotional responses to perceived threats to safety and continuity (Greenberg et al., 1997; Pihkala, 2018). Together, these perspectives suggest that eco‐emotions are different responses to environmental crisis and may affect mental health and wellbeing in different ways.

Emerging adulthood encompasses individuals between the ages of 18–29 years (Arnett, 2014). This is a stage in life where individuals seek to establish social and intimate relationships and develop skills for their professional and social lives. It is characterized by an aspirational, ambitious outlook and a tendency to strive to achieve one's goals. It has been proposed to be a formative stage on which the foundation of one's remaining adult life rests (Arnett, 2014; Erikson & Erikson, 1998). Younger adults tend to report elevated scores of eco‐anxiety, eco‐fear, and eco‐hopelessness (Gibson et al., 2020; Heeren et al., 2022; Kühner et al., 2025). Rather than looking forward to a life filled with aspirations and hopes, younger generations are being left with a life that will be significantly impacted by environmental crises. The perceived apathy and disavowal displayed by community and national leaders furthers the sense of distrust and hopelessness younger generations feel when thinking about their future in a world ravaged by anthropogenic environmental degradation (Galway & Field, 2023; Hickman et al., 2021). Having to face the consequences of the environmental crisis can have a deep impact on their ability to develop into functional, well‐adjusted individuals, with younger generations being the most vulnerable to the mental health and wellbeing impacts of the climate and ecological crises (Clayton & Karazsia, 2020; Hajek & Konig, 2023; Heeren et al., 2022; Kühner et al., 2025; Ojala, 2012; Searle & Gow, 2010). Therefore, understanding the impact of eco‐emotions on the emotional wellbeing of young adults will help mental health professionals provide relevant support to build resilience and adaptability to cope with eco‐emotions (Galway & Field, 2023; Heeren et al., 2022; Hickman et al., 2021; Nadarajah et al., 2022).

### Objectives

This systematic review aims to examine the prevalence of eco‐emotions among young adults and to conduct a meta‐analysis of the association between eco‐emotions and mental health and well‐being. Specifically, it seeks to (1) determine the levels of eco‐emotions experienced by young adults aged 18–29, and (2) assess the relationship between eco‐emotions and mental health and wellbeing within this age group.

## METHODS

A systematic review and meta‐analysis were conducted in accordance with Cochrane guidelines (Higgins et al., 2023) and reported following the PRISMA statement (Page et al., 2021), using the structure outlined by Tunç et al. (2023).

### Protocol

The protocol was registered and published on the PROSPERO database (Registration ID: CRD42023396117): https://www.crd.york.ac.uk/PROSPERO/view/CRD42023396117.

### Eligibility criteria

Studies were included if they: a) were published in English, Nepali, or Hindi; b) report quantitative data on eco‐emotions experienced by young adults (aged 18–29); c) report correlation *r* data between eco‐emotions and mental health and/or wellbeing. If a study had data from participants that extended beyond the target age group, it was included if 50% or more of the participants were within the 18–29 target age group, due to the paucity of studies conducted solely on the target population. Where possible, authors were contacted and asked to provide data by age group.

The term “eco‐emotions” was defined as any emotion experienced by individuals due to their perception, attitudes, beliefs, and concerns of climate and/or ecological crises. The climate and ecological crises were defined as any and all impacts due to global warming/climate change, or other anthropogenic environmental degradation.

To address the first review question, levels of eco‐emotions reported in studies were reviewed. To answer the second review question, Pearson's zero‐order correlation *r* was obtained to assess associations between eco‐emotions and mental health and/or wellbeing outcomes, and a meta‐analysis was conducted.

Case studies, books, book chapters, qualitative studies, and studies without sufficient details to enable critical appraisal were excluded. Cross‐sectional and longitudinal studies were included, including conference abstracts and unpublished studies, theses, etc., where sufficient details were available either in the original content or via contacting the author(s) of those studies. Where studies were longitudinal, data were extracted from baseline data where possible, or alternatively from the wave that aligned with the target age group specified for the review.

### Search strategy

The following databases were searched via the Ovid platform: AMED (Allied and Complementary Medicine), CAB Abstracts, APA PsycInfo, Books@Ovid, Journals@Ovid Full Text, Your Journals@Ovid, APA PsycArticles Full Text, CAB Abstracts, Embase Classic+Embase, Global Health, Ovid MEDLINE(R), and Epub Ahead of Print, In‐Process, In‐Data‐Review & Other Non‐Indexed Citations, Daily and Versions. To capture unpublished and grey literature, additional searches were conducted through Google Scholar and OpenSIGLE. Additionally, backward citation tracking (screening the reference lists of all included studies) and forward citation tracking (by using Google Scholar to identify studies that cited included papers) were conducted to ensure comprehensive coverage, including potentially unpublished or non‐peer‐reviewed sources. Where the full text of an article deemed to meet the inclusion criteria was not accessible, corresponding authors were contacted to request access to the full papers. Literature search began in December 2022 and was updated in November 2024 using the same search and study selection strategy. No limit was set for the search start date.

The following search terms and phrases related to eco‐emotions were used to catch relevant studies:

(“ecoanxi*” or “eco* anxi*” or “climate anxi*” or “climate crisis” or “eco* emotion*” or “eco* depress*” or “eco* anger*” or “eco* worry*” or “eco* fear*” or “eco* hopelessness*” or “eco* hope” or “climate emotion*” or “solastalgi*” or “climate distress” or “eco* distress”) AND (adolescen* or teen* or student* or “young adult*” or “young people” or “undergraduate*” or “emerging adult*”) AND (“mental wellbeing” or “mental health” or mental well‐being” or “anxi*” or “depress*” or “stress” or “anger” or “fear” or “psycholo* well‐being” or “life satisfaction” or “positive emotion*”).

The database search was conducted via the OVID platform using the predefined search terms across the aforementioned databases.

### Study selection

A total of 1773 studies were obtained through an Ovid database search. Duplicates were removed using Ovid's deduplication function. After removing duplicates, 1,187 studies remained. Studies obtained through other methods (e.g. grey literature, unpublished data) yielded an additional eight studies. In total, 1,195 studies were obtained for screening.

**FIGURE 1:**
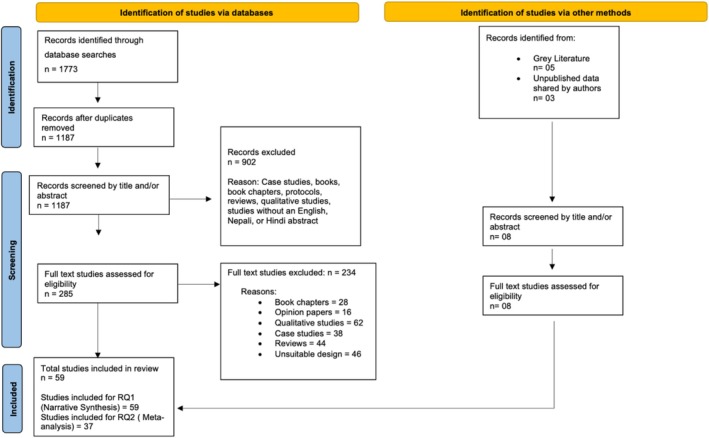
PRISMA flow chart detailing the number of studies considered at each stage of the selection process. N: number of records.

Titles and abstracts were reviewed and extracted by the first author. A total of 902 irrelevant records were excluded based on title and abstract screening, resulting in 293 publications retrieved for full‐text review and eligibility assessment. Details can be found in Figure 1.

In the full‐text review stage, studies that reported levels of eco‐emotions with the target population were included for review question one, which was addressed through narrative synthesis of data. Studies that conducted correlation analysis (i.e. Pearson's *r*) between eco‐emotions and mental health and/or mental wellbeing were included to address review question two via meta‐analysis. Twenty‐three studies examined both eco‐emotions and mental health and/or wellbeing but did not report correlation coefficients between variables. To obtain the necessary data, the corresponding authors of those studies were contacted. One author (Hogg et al., 2024 study 1; 2024 study 2) shared two datasets; Leviston et al. (2021) shared one unpublished dataset. Wullenkord et al. (2021); Larionow et al. (2022); Contreras et al. (2023); Ediz and Yanik (2023); Heinzel et al. (2023); Prencipe et al. (2023); Cimsir et al. (2024) responded to requests to share data that was suitable for the current review.

In total, 59 studies, including one unpublished dataset, were included in the review.

### Data extraction

A data extraction form was used to extract the following details: Author(s), year of publication, country, participants' age, sample size, recruitment method, study design and methodology, measures used, levels of eco‐emotions; relationships between eco‐emotions on mental health and/or wellbeing, and statistical analysis of relevant outcome measures. Where a study included more than one relevant measure, details for all relevant measures were extracted.

### Risk of bias (quality) assessment

Quality assessment focused on methodological risk of bias assessment. A risk of bias (RoB) tool modeled on Morris (2017) and Tunç et al. (2023) was adapted specifically for this review. Existing quality assessment tools were not deemed suitable, as they focused on quality of reporting only; reporting based on methodological quality was missing, thereby necessitating the adaptation of the RoB tool. The RoB tool consists of six domains: sampling and recruitment, sampling size, eco‐emotions measurement, mental health measurement, mental wellbeing measurement, and statistical analysis. Studies were rated as “well covered,” “adequately addressed,” “poorly addressed,” “not reported,” or “not applicable” for each domain, along with a summary domain. The sampling, recruitment, and measurement domains were adapted from the Appraisal tool for Cross‐Sectional Studies (AXIS; Downes et al., 2016). Due to numerous ad‐hoc eco‐emotions measurement scales in the literature, it was decided to rate a study as “adequately addressed” if suitable psychometrics were reported for their ad‐hoc eco‐emotions measurement scales.

Two reviewers independently assessed all studies, and then the evaluations were compared. Discrepancies were resolved through discussion among the review team until consensus was achieved.

### Statistical analyses for meta‐analyses

All statistical analyses were run in R software (R Core Team, 2023) using R Studio (Rstudio, 2023) and guided by Harrer et al. (2021). The following packages were used: Meta (Schwarzer, 2007), psych (Revelle, 2017), tidyverse (Wickham et al., 2019), and dmetar (Harrer et al., 2019).

Random‐effects modeling was used to synthesize observed effects into a pooled effect size, as included studies were not from a homogeneous population. Fisher's z transformation of correlations was calculated automatically by R packages. The correlation coefficient effect sizes were categorized as small, medium, and large for 0.10, 0.30, and 0.50 effect sizes, respectively (Cohen, 2013). Restricted maximum likelihood estimator (Viechtbauer, 2005) was utilized to estimate between‐study heterogeneity variance (*τ*2). Finally, *Q*‐statistics, *I* ^2^, and prediction intervals were calculated to investigate between‐study heterogeneity.

## RESULTS

Fifty‐nine studies reporting levels of eco‐emotions were included in addressing review question one. Thirty‐seven studies reported correlation values between eco‐emotions and mental health and were included for the meta‐analysis.

The review highlighted the broad way in which eco‐anxiety has been defined in the literature, with different scales operationalizing the construct in different ways. To address these differences, this review took mean and correlation scores from the cognitive‐emotional subscale of the climate‐anxiety scale (Clayton & Karazsia, 2020) and from the affective subscale of the Hogg eco‐anxiety scale (Hogg et al., 2021) respectively, as these were deemed the subscales that had the greatest correspondence with the ’emotional aspect’ of experiencing eco‐anxiety.

Due to insufficient evidence being available on other eco‐emotions (i.e. eco‐anger, eco‐fear, eco‐hope, eco‐guilt, etc.), meta‐analysis was conducted solely on eco‐anxiety.

### Characteristics of included studies

The total number of participants was 65,997 across all 39 studies. Studies included in the review are outlined in Table A.1.1 of the Supplementary Appendix. The largest participant sample from a single study was 12,246; and the smallest was 31. Biological sex was given for the majority of studies, and heavily skewed towards females (63.16% on average). The majority of participants were university students. Studies were mostly conducted in high‐income countries, albeit studies from low‐to‐middle‐income countries were emerging (Figure A.2.1 of the Supplementary Appendix). We categorized studies as multinational where participants were drawn from two or more geographically dispersed countries. Studies were primarily published in peer‐reviewed journal articles. One was reported in a Master's dissertation, and one in a PhD thesis. Figure A.2.2 of the Supplementary Appendix illustrates publication of studies over time.

### Risk of bias (quality) assessment of the included studies

The quality of 59 studies was assessed using the developed quality assessment tool. All studies were independently assessed by two raters, with an initial overall consistency of 84%. Domains on which reviewers initially disagreed were systematically discussed until consensus was achieved. A traffic light plot was used to visually summarize risk of bias ratings across domains and individual studies (Figure A.3.1 of the Supplementary Appendix).

Of 59 studies assessed in relation to review question one, 10 were assessed as having an overall high risk of bias, with the other 49 rated as having an overall low risk of bias. Of the 10 studies rated as high risk of bias, one (Gibson et al., 2020) had a low sample size in the age‐range 18–29 and likely low statistical power for the current purpose (i.e. domain 2). Their study had a larger overall sample, but this review included only participants aged 18–29. For the same reason, sample sizes in eight other studies (Cimsir et al., 2024; Contreras et al., 2023; Hajek & Konig, 2023; Heeren et al., 2022; Heinzel et al., 2023; Larionow et al., 2022; Tam et al., 2023; Wullenkord et al., 2021) were lower in the present review than the full samples in those studies. These studies have been marked with an asterisk symbol “*” in Figure A.3.1 of the Supplementary Appendix.

Cimsir et al. (2024); Lutz (2023 study 1; 2023 study 2; 2023 study 3; 2023 study 5) were rated as high risk of bias due to the sample being derived solely from university students, thus likely being non‐representative (i.e. domain 1); while Prencipe et al. (2023); Sciberras and Fernando (2022); Swim et al. (2022) and Yatirajula et al. (2023) were deemed to have a poorer quality of assessment tool(s) used to measure eco‐emotions (domain 3). Eco‐emotions is a relatively new area; validated tools and scales to measure eco‐emotions are sparse, leading to ad‐hoc scales being used that often lack strong psychometric properties. Despite limitations, these studies helped address the first review question and were included in the review. The summary graph of the proportion of studies with risk‐of‐bias judgments within each domain is presented in Figure A.3.2 of the Supplementary Appendix. Summary graphs were weighted by the number of participants (*N*) (Higgins et al., 2023).

To address the second review question, the quality of 37 studies was assessed using the same quality assessment tool. Each of the 37 studies was evaluated across seven different domains in total (six main domains and one overall RoB assessment domain). An overall consistency of 82% was achieved in the RoB assessment for the total of 259 domains. Domains on which reviewers initially disagreed were systematically discussed until consensus was achieved. A traffic light plot was used to visually summarize risk of bias ratings across domains and individual studies (Figure A.3.3 of the Supplementary Appendix).

Out of the 37 studies included to address review question two, five studies were assessed as having an overall high risk of bias, while the remaining studies were rated as having an overall low risk of bias. Cimsir et al. (2024) and Lutz et al. (2023 study 2; 2023 study 3; 2023 study 5) were rated as having a high risk of bias due to the sample being derived solely from university students, thus likely being non‐representative (i.e. domain 1). Prencipe et al. (2023) reported on the poor quality of assessment tool(s) used to measure eco‐emotions (domain 3). The summary graph of the proportion of studies with risk‐of‐bias judgments within each domain is presented in Figure A.3.4 of the Supplementary Appendix. Summary graphs were weighted by the number of participants (*N*) (Higgins et al., 2023).

### Narrative synthesis of results

The study of eco‐emotions, being a relatively new field in mental health research, has only a few validated scales available (Table A.4.1 of the Supplementary Appendix).

Eco‐anxiety was expressed through measurement of levels of current and future anxiety, worry, and concern for climate and ecological crises. The review emphasized the diverse ways in which eco‐anxiety has been defined and measured in the existing literature, reflecting the evolving nature of the construct. The climate‐anxiety scale extends beyond “emotion” by focusing on rumination to measure climate anxiety based on cognitive‐emotional and functional impairment aspects. In comparison, the Hogg eco‐anxiety Scale views eco‐anxiety multidimensionally, seeking to measure the frequency of eco‐anxiety over a two‐week period via affect and rumination. The climate‐change worry scale operationalized climate‐change worry based on ‘proximal and personal worry about climate change’ (Stewart, 2021 Pg. 4). Verplanken (2020 study 1; 2020 study 2; 2020 study 3) operationalized global warming worry based on a list of worries shared by participants to create emotion clusters related to habitual worry about global warming. Finally, Lutz (2023 study 1; 2023 study 2; 2023 study 3) adopted a four‐item scale measuring ecological stress (Helm et al., 2018) that operationalized the construct based on perceptions of environmental problems.

All 59 reviewed studies reported levels of eco‐anxiety. Table A.4.2 of the Supplementary Appendix shows the average and weighted by *N* mean scores of studies based on the eco‐anxiety scale.

#### Prevalence of eco‐emotions

As some measures focused on the frequency of eco‐emotional experience and others on their intensity, mean scores are presented descriptively and interpreted within the context of each specific scale.

Importantly, this review does not impose uniform cut‐off points to define “low,” “moderate,” or “high” levels of eco‐emotions across scales, as such categorizations would be arbitrary without validated interpretative guidelines. When available, scale‐specific norms or theoretical anchors from the original scale developers are noted. Otherwise, scores are considered in relation to other studies using the same measure to support contextual interpretation.

##### Levels of eco‐anxiety

The climate‐anxiety scale (Clayton & Karazsia, 2020) was the most frequently utilized measure across the reviewed studies. This scale assesses the general frequency of climate‐anxiety‐related experiences with items rated from 1 (“Never”) to 5 (“Almost always”). In most studies, reported mean scores fell toward the lower end of the scale range (e.g., Clayton & Karazsia, 2020 study 1; 2020 study 2; Heeren et al., 2022; McBride, 2022; Whitmarsh et al., 2022; Wullenkord et al., 2021; Hajek & Konig, 2023; Fekih‐Romdhane et al., 2024), although several studies reported comparatively higher scores, often in contexts where recent climate‐related disasters or acute environmental concerns may have heightened public climate‐anxiety (Abou Jaoude et al., 2024; Reyes et al., 2021; Contreras et al., 2023; Ediz & Yanik, 2023; Simon et al., 2022; Tam et al., 2023). However, in the absence of validated interpretative thresholds, these scores are reported descriptively and compared across studies using the same instrument.

In contrast, the Hogg eco‐anxiety (Hogg et al., 2021) employs a different framework, asking respondents how often, over the past two weeks, they have been “bothered” by problems when thinking about environmental crises. Response options range from 0 (“Not at all”) to 3 (“Nearly every day”). This time‐bound approach aligns more closely with screening tools for generalized anxiety. Mean scores across studies using this scale varied, with some reporting higher averages (e.g., Hogg et al., 2024 study 1; 2024 study 2; Lutz, Zelenski, & Newman, 2023; Sampaio et al., 2023; Er et al., 2024), and others reporting relatively lower scores (e.g., Hogg et al., 2021 study 1; 2021 study 2; Heinzel et al., 2023; Cimsir et al., 2024; Rodriguez et al., 2024). As with the climate‐anxiety scale, these findings are presented for within‐measure comparison rather than cross‐scale comparison.

Additionally, several studies employed alternative measures, including the climate‐change distress scale (Kulcar et al., 2022; Searle & Gow, 2010), the climate‐change worry scale (Stewart, 2021 study 3), and a range of ad‐hoc instruments. These ad‐hoc scales vary in format and response options, limiting interpretability and comparability. Therefore, while some studies reported average scores above or below their respective scale midpoints (e.g., Hickman et al., 2021; Lawrance et al., 2022; Ogunbode et al., 2021; Stanley et al., 2021), such values should be interpreted with caution unless supported by normative data or theoretical anchors.

##### Levels of eco‐anger

All studies reporting eco‐anger levels employed ad‐hoc measures, often without standardized cut‐offs or normative benchmarks. In two studies, (Leviston et al., 2021; Stanley et al., 2021) eco‐anger was assessed using a 0–100 sliding scale (“0 = not at all this way 100 = a great deal”). Both studies reported mean scores of 53.35 and 56.48, respectively. Swim et al. (2022) employed a one‐item ad‐hoc measure rating intensity of eco‐anger on a four‐point scale from 0 “not at all” to 3 “very.” Their younger sample reported a mean score of 1.23. This score falls in the lower‐midpoint of the scale, which may tentatively reflect low‐to‐moderate levels when interpreted relative to the scale's midpoint, though this should be treated with caution given the absence of validated thresholds. Similarly, Hickman et al. (2021) found 56.8% of their participants reported eco‐anger using an ad‐hoc one‐item dichotomous scale (Yes/No) with an additional option for non‐response (“Prefer not to say”). Furthermore, studies linked eco‐anger with greater pro‐environmental behaviors (Verplanken et al., 2020 study 3; Stanley et al., 2021).

##### Levels of eco‐fear

Gibson et al. (2020) assessed eco‐fear using both quantitative and qualitative methods. Quantitatively, participants rated their eco‐fear on an ad‐hoc four‐point scale ranging from 1 (“Not at all”) to 4 (“Extremely”), with a reported mean score of 2.04. While no standardized cut‐off scores were provided, this score falls slightly above the midpoint of the scale, suggesting moderate levels of eco‐fear. This interpretation was further supported by qualitative responses describing high emotional intensity. Hickman et al. (2021) and Galway and Field (2023) found 67.3% and 66% of participants reported eco‐fear, respectively, using a similar ad‐hoc one‐item dichotomous scale, especially in response to perceived environmental crises, lack of global action, and personal helplessness. Helplessness, hopelessness, and powerlessness related to perceived environmental threats were also reported (Galway & Field, 2023; Gibson et al., 2020; Hickman et al., 2021; Kulcar et al., 2022; Lawrance et al., 2022).

##### Levels of eco‐guilt, eco‐sadness, and eco‐depression

Hickman et al. (2021), Swim et al. (2022), and Galway and Field (2023) reported eco‐guilt and eco‐sadness based on mean responses to single‐item ad‐hoc scales. Similarly, eco‐depression was reported by Stanley et al. (2021), Leviston et al. (2021), Hickman et al. (2021), and Galway and Field (2023).

Notably, in the two studies that investigated perceptions of government responses to climate change, a substantial majority of participants reported feelings of betrayal (Galway & Field, 2023; Hickman et al., 2021), with highest levels reported in Brazil, India, the Philippines, and Australia (Hickman et al., 2021). Interpretation of these findings is limited due to the use of ad‐hoc measures without normative benchmarks. As such, findings are presented descriptively and should be interpreted with caution.

##### Levels of eco‐hope

Lastly, Hickman et al. (2021); Lawrance et al. (2022), and Galway and Field (2023) reported on eco‐hope/optimism, typically finding a low score based on average responses to Likert‐scale items. Notably, one study reported positive associations between eco‐determination and pro‐environmental behaviors (Verplanken et al., 2020 study 3).

#### Results of meta‐analysis

Due to the insufficiency of studies involving other eco‐emotion measures or mental wellbeing outcomes, meta‐analyses focused on studies that presented correlation data between eco‐anxiety and constructs of anxiety, depression, and/or stress.

##### Meta‐analysis of relationship between eco‐anxiety and depression

**FIGURE 2:**
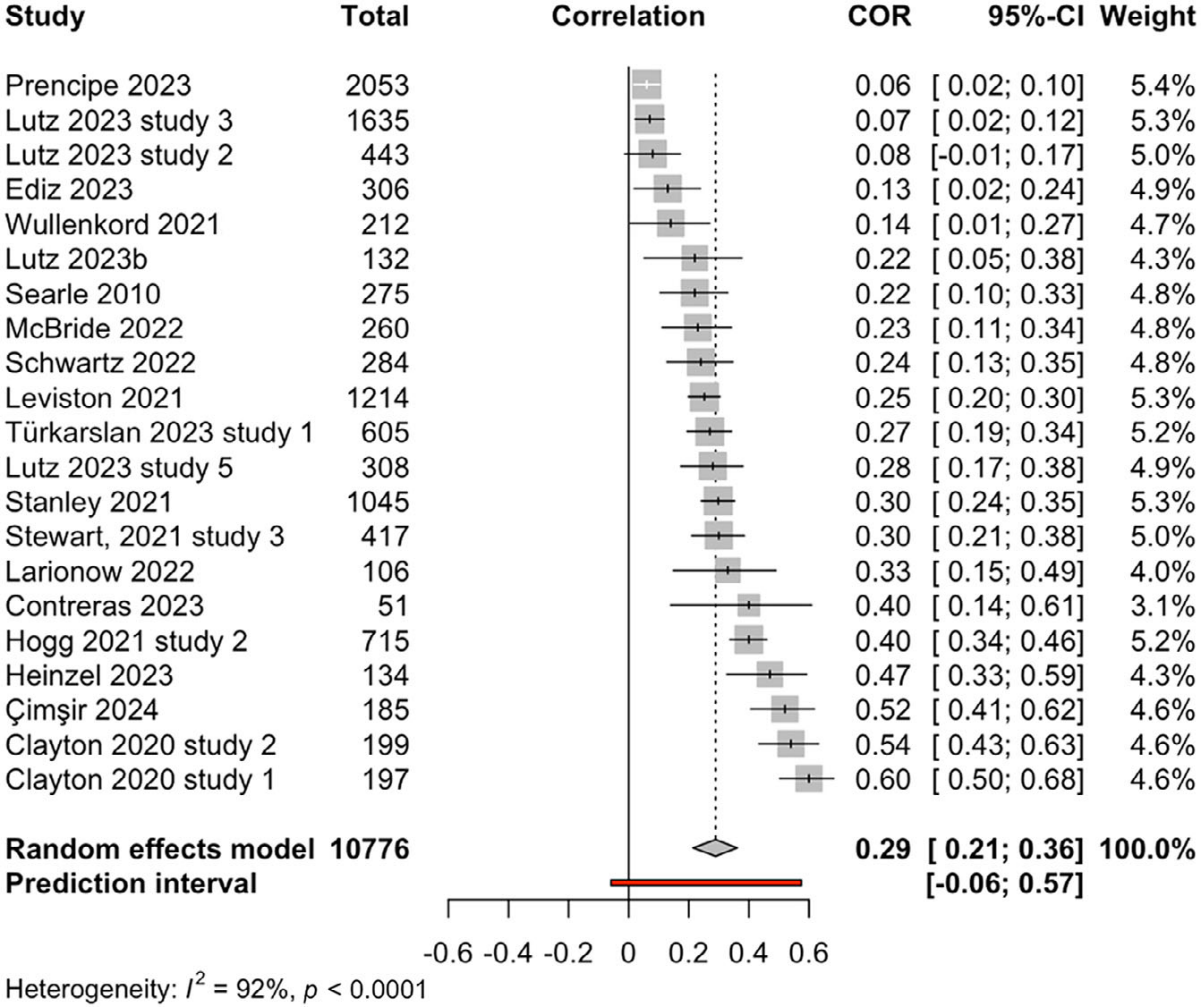
Forest plot of relationship between eco‐anxiety and depression. *Note:* *r* = 0.29, 95%CI [0.21; 0.36], *p* < .0001.

Meta‐analysis based on 21 studies was conducted to examine the association between eco‐anxiety and depression (see forest plot in Figure 2). The total number of participants was 10,776. The results showed a significant moderate effect size relationship between eco‐anxiety and depression, with a positive pooled correlation *r* = 0.29, 95%CI [0.21; 0.36], *p* < .0001, *k* = 21, *o* = 10,776.

##### Meta‐analysis of relationship between eco‐anxiety and anxiety

**FIGURE 3:**
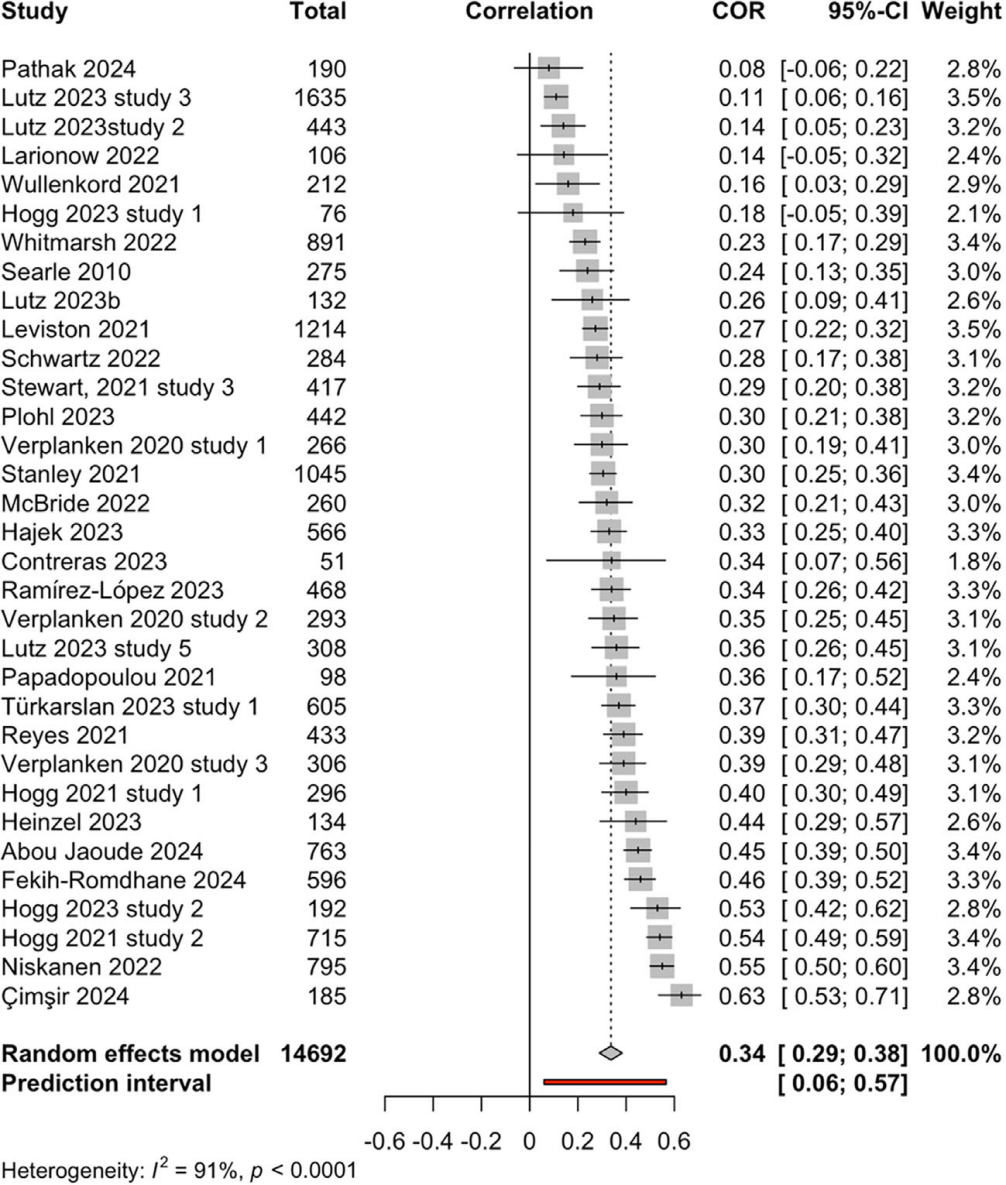
Forest plot of relationship between eco‐anxiety and anxiety. *Note:* *r* = 0.34, 95%CI [0.29; 0.38], *p* < .0001.

Meta‐analysis based on 33 studies was conducted to examine the association between eco‐anxiety and anxiety (see forest plot in Figure 3). The total number of participants was 14,692. The results showed a significant moderate effect size relationship between eco‐anxiety and anxiety, with a positive pooled correlation *r* = 0.34, 95%CI [0.29; 0.38], *p* < .0001, *k* = 33, *o* = 14,692.

##### Meta‐analysis of relationship between eco‐anxiety and stress

**FIGURE 4:**
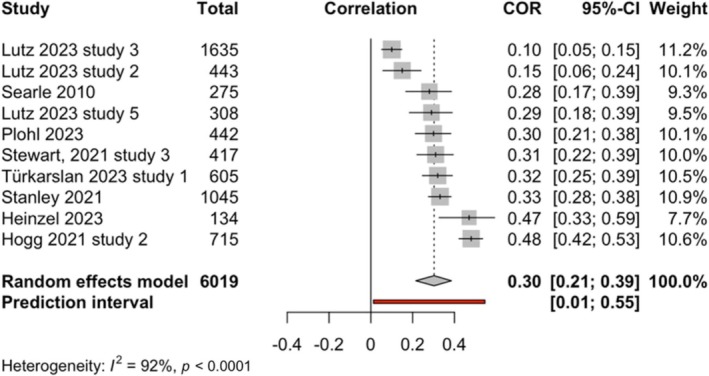
Forest plot of relationship between eco‐anxiety and stress. *Note:* *r* = 0.30, 95%CI [0.21; 0.39], *p* < .0001.

Meta‐analysis based on 10 studies was conducted to examine the association between eco‐anxiety and stress (see forest plot in Figure 4). The total number of participants was 6,019. The results showed a significant moderate effect size relationship between eco‐anxiety and stress, with a positive pooled correlation *r* = 0.30, 95%CI [0.21; 0.39], *p* < .0001, *k* = 10, *o* = 6,019.

#### Tests for heterogeneity

All eligible studies were included, encompassing diverse populations and methodologies. Heterogeneity tests were conducted to see if effect sizes in the meta‐analyses were reasonably consistent or whether they varied.

##### Heterogeneity for relationship between eco‐anxiety and depression

Q‐statistic was significant, indicating that studies in the meta‐analysis of the relationship between eco‐anxiety and depression did not exhibit a common effect size, *Q*(20) = 257.53 *p* < 0.0001. Between‐study heterogeneity was estimated at Tau^2^ = 0.03 (95%CI: 0.01–0.06); with *I* ^2^ value of 92% (95%CI: 90–94%). This indicates that 92% of the variance in observed effect sizes was attributable to true heterogeneity between studies, rather than random sampling error. The prediction interval ranged from *g* = −0.06 to 0.57, indicating that negative correlations cannot be ruled out for future studies. Therefore, it is anticipated that in future studies, the true effect size in 95% of populations comparable to those in this analysis will fall in this prediction interval range (−0.06 to 0.57).

##### Heterogeneity for relationship between eco‐anxiety and anxiety

Q‐statistic was significant, indicating that studies in the meta‐analysis of the relationship between eco‐anxiety and anxiety did not exhibit a common effect size, *Q*(32) = 341.94, *p* < 0.0001. Tau^2^ was estimated at 0.02 (95% CI: 0.01–0.04). *I* ^2^ statistic was 91% (95% CI: 88–93%). This indicates that 91% of the variance in observed effect sizes was attributable to true heterogeneity between studies, rather than random sampling error. Prediction interval ranged from *g* = 0.06 to 0.57, suggesting that positive correlations cannot be ruled out for future studies. Therefore, it is anticipated that the true effect size in 95% of populations comparable to those in this analysis will fall in this prediction interval range (0.06 to 0.57) in future studies. The prediction interval does not cross 0; therefore, it can be predicted that most studies would find a positive relationship between eco‐anxiety and anxiety.

##### Heterogeneity for relationship between eco‐anxiety and stress

Q‐statistic was significant, indicating that studies in the meta‐analysis of the relationship between eco‐anxiety and anxiety did not exhibit a common effect size, *Q*(9) = 115.99, *p* < 0.0001. Between‐study heterogeneity was estimated at Tau^2^ = 0.02 (95%CI: 0.01–0.06); *I* ^2^ value was 92% (95%CI: 88–95%). Prediction interval ranged from *g* = 0.01 to 0.55, suggesting that positive correlations cannot be ruled out for future studies. Therefore, it is anticipated that the true effect size in 95% of populations comparable to those in this analysis will fall in this prediction interval range (0.01 to 0.55) in future studies. Since the prediction interval does not cross 0, it can be expected that most future studies are likely to observe a positive association between eco‐anxiety and stress.

#### Sensitivity analyses

Sensitivity analyses were conducted in order to explore whether ad hoc scales influenced the meta‐analytic results. Removing ad hoc scales slightly increased the effect size for the relationship between eco‐anxiety and depression and had no notable effect on the relationship between eco‐anxiety and either anxiety or stress. Results can be found in Section A5 of the Supplementary Appendix.

#### Outliers and influential cases

Due to a high level of heterogeneity, outliers and influential cases were sought out. If the 95% confidence interval of a study's effect size does not overlap with the pooled effect size's confidence interval, it can be categorized as an outlier (Harrer et al., 2021). Accordingly, pooled correlations were recalculated after removing outliers from the meta‐analyses.

In the meta‐analysis of the relationship between eco‐anxiety and depression, seven studies (Clayton & Karazsia, 2020 study 1; 2020 study 2; Lutz, Passmore, et al., 2023 study 2; and 2023 study 3; Prencipe et al., 2023; Cimsir et al., 2024; and Hogg et al., 2021 study 2) were identified as outliers and removed, and heterogeneity was tested again. The pooled effect size decreased marginally, by 0.03, while the prediction interval range reduced significantly, and *I* ^2^ statistic dropped to 42%. The recalculated meta‐analysis results are presented in Table 1.

**TABLE 1:** Outlier analyses for meta‐analysis of relationship between eco‐anxiety and depression, anxiety, and stress.

Analysis	*k*	*r*	95% CI	*p*	I^2^	95%PI
**Eco‐anxiety‐depression**						
• *Main analysis*	21	0.29	0.21; 0.36	< .0001	92%	−0.06; 0.57
• *results with outliers removed*	14	0.26	0.22; 0.30	< .0001	42%	0.18; 0.34
**Eco‐anxiety‐anxiety**						
• *Main analysis*	33	0.34	0.29; 0.38	< .0001	91%	0.06; 0.57
• *results with outliers removed*	23	0.32	0.29; 0.34	< .0001	34%	0.24; 0.39
**Eco‐anxiety‐stress**						
• *Main analysis*	10	0.30	0.21; 0.39	< .0001	92%	0.01; 0.55
• *results with outliers removed*	07	0.32	0.28; 0.36	< .0001	0%	0.28; 0.36

In the meta‐analysis of the relationship between eco‐anxiety and anxiety, 10 studies (Hogg et al., 2021 study 2; and 2024 study 2; Lutz, Passmore, et al., 2023 study 2; and 2023 study 3; Wullenkord et al., 2021; Fekih‐Romdhane et al., 2024; Niskanen 2022; Pathak, 2024; Abou Jaoude et al., 2024; and Cimsir et al., 2024) were identified as outliers and removed, and heterogeneity was tested again. The pooled effect size decreased marginally, by 0.02; while the prediction interval range reduced significantly, and *I* ^2^ statistic dropped to 34%. The recalculated meta‐analysis results are presented in Table 1.

In the meta‐analysis of the relationship between eco‐anxiety and stress, three studies (Lutz, Passmore, et al., 2023 study 2; 2023 study 3; and Hogg et al., 2021 study 2) were identified as outliers and removed, and heterogeneity was tested again. The pooled effect size increased marginally, by 0.02; while the prediction interval range reduced, and the *I* ^2^ statistic dropped to 0%. The recalculated meta‐analysis results are presented in Table 1.

Lutz et al. (2023 study 2; 2023 study 3); and Hogg et al. (2021 study 2) were identified as outliers in all of the meta‐analyses. No shared characteristics were found among the studies identified as outliers. Variations were mainly observed in sample characteristics, assessment tools, and risk of bias status. Consequently, interpreting the results post‐exclusion of these studies requires caution, given that different methods for identifying outliers may lead to varying conclusions.

Influence analyses were performed to identify studies that most influenced results, as certain studies can substantially affect the pooled effect size and heterogeneity. A leave‐one‐out approach was adopted (Harrer et al., 2021). A study's influence on the pooled result and overall heterogeneity (based on *Q*‐statistic) was illustrated with the help of Baujat plots (Baujat et al., 2002) for eco‐anxiety and depression (Figure A.6.1 of the Supplementary Appendix), eco‐anxiety and anxiety (Figure A.6.2 of the Supplementary Appendix), and eco‐anxiety and stress (Figure A.6.3 of the Supplementary Appendix).

#### Meta‐regression (moderator) analyses

Potential moderators were identified, and meta‐regression analyses were conducted to explore sources of heterogeneity. No statistically significant moderator variables were found for the relationships between eco‐anxiety and depression, eco‐anxiety and anxiety, nor eco‐anxiety and stress. Details of the results can be found in Section A7 of the Supplementary Appendix.

#### Publication bias

Contour‐enhanced funnel plots were created for meta‐analyses of relationships between eco‐anxiety and depression (Figure A.8.1 of the Supplementary Appendix), eco‐anxiety and anxiety (Figure A.8.2 of the Supplementary Appendix), and eco‐anxiety and stress (Figure A.8.3 of the Supplementary Appendix). If there is no publication bias, the data in the funnel plots should create a roughly symmetrical shape. Moreover, Egger's regression tests (Egger et al., 1997) were performed to identify asymmetry (Table 2). Egger's test did not detect any asymmetry in the funnel plots for the relationships between eco‐anxiety and anxiety or eco‐anxiety and stress. The test indicated significant funnel plot asymmetry in the analysis of the relationship between eco‐anxiety and depression, suggesting the presence of potential publication bias.

**TABLE 2:** Egger's regression results for testing asymmetry in the funnel plots for eco‐anxiety and depression; anxiety; and stress.

Analysis	*Intercept*	*95% CI*	t	*p*
*Depression*	4.49	1.57; 7.42	3.01	.007
*Anxiety*	1.10	−1.95; 4.14	0.71	.486
Stress	4.62	−1.94; 11.18	1.38	.205

To adjust the asymmetry in the funnel plot of the relationship of eco‐anxiety and depression, the trim and fill method was used (Duval & Tweedie, 2000). This technique imputes missing effects to the plot to achieve symmetry and subsequently recalculates the pooled effect for the meta‐analysis. The contour‐enhanced funnel plot with the trim and fill method is shown in Figure A.8.4 of the Supplementary Appendix. Eight missing results with small effect sizes than the observed effect sizes were added to the funnel plot. The recalculated pooled correlation for the relationship between eco‐anxiety and depression decreased to 0.18, and the *I* ^2^ statistic increased by 2% to 94%. The prediction interval range expanded to −0.34 to 0.61. This finding suggests that even after applying the trim and fill method, the possibility of observing either a positive or negative relationship in future studies cannot be excluded. Results with eight added studies are shown in Table 3.

**TABLE 3:** Results of the trim and fill method for eco‐anxiety and depression.

Analysis	*k*	*r*	95% CI	*p*	I^2^	95%PI
*Main analysis*	21	0.29	0.21; 0.36	< .0001	92%	−0.06; 0.57
*Trim and fill (eight studies added)*	29	0.18	0.08; 0.27	< .0011	94%	−0.34; 0.61

## DISCUSSION

This review systematically examined the literature on eco‐emotions experienced by young adults in order to answer two questions: the levels of eco‐emotions reported, and the relationship between eco‐emotions and mental health. The literature was scarce on the relationship between eco‐emotions and mental health outcomes, apart from eco‐anxiety. Our findings suggested positive relationships between eco‐anxiety and anxiety, depression, and stress. Our findings of negative eco‐emotions being reported were expected. Many young adults feel these negative eco‐emotions intensely and frequently, with literature demonstrating that younger populations were significantly affected by the climate and ecological crises and are most vulnerable to its impact on mental health (Clayton & Karazsia, 2020; Hajek & Konig, 2023; Heeren et al., 2022; Searle & Gow, 2010). These eco‐emotions and effects on mental health may be exacerbated by beliefs that preventative and preservative actions are lacking and that they will live a significant portion of their lives facing the impacts of the climate and ecological crises (Diffey et al., 2022; Hickman et al., 2021; Thompson et al., 2022).

### Levels of eco‐emotions

Eco‐anxiety was the most commonly reported eco‐emotion. Although a wide range of scales (including many ad‐hoc measures) were used to operationalize eco‐anxiety, studies consistently found higher scores within their respective measures in regions directly affected by climate and ecological crises. Hickman et al. (2021) reported a higher prevalence of eco‐anxiety and eco‐fear among participants from the Philippines, India, Nigeria, and Australia relative to those from other countries. Similarly, studies using the climate‐anxiety scale reported higher scores in regions that had recently experienced natural disasters. Several more recent studies reported higher scores within their respective scales, suggesting that increased climate and ecological impacts globally may be contributing to experiences of eco‐anxiety. As natural disasters due to anthropogenic environmental degradation are expected to increase, alongside the intensification of socioeconomic instability, eco‐anxiety is likely to increase among populations, particularly where there is a lack of support to help cope and build resilience (IPCC, 2023). This is likely to have a detrimental effect on the overall mental health of young adults. In particular, perceived powerlessness in the face of global inaction has been linked to psychological distress in the group (Pihkala, 2018).

Two studies reported moderate average scores of eco‐anger (Leviston et al., 2021; Stanley et al., 2021). Interestingly, studies consistently found that eco‐anger was positively associated with pro‐environmental behaviors (Verplanken et al., 2020 study 3; Stanley et al., 2021). This may reflect the energizing aspect of anger that can make people feel stronger and powerful (Lerner et al., 2003; Lerner & Keltner, 2001). In this way, eco‐anger may serve an adaptive function, enabling individuals to channel distress into action. By promoting engagement and agency, eco‐anger may also mitigate feelings of eco‐anxiety, eco‐helplessness, and eco‐powerlessness, especially when individuals perceive avenues for collective or personal action (Stanley et al., 2021; Stanley et al., 2025). This aligns with theoretical perspectives that conceptualize anger as an approach‐oriented emotion linked to perceived injustice and motivation for action (Lerner & Keltner, 2001).

Across studies, low eco‐hope or optimism were reported based on participants' average scores on Likert‐type items (e.g., Galway & Field, 2023; Hickman et al., 2021; Lawrance et al., 2022). While normative thresholds were not available, these scores tended to fall toward the lower end of their respective scale ranges, suggesting comparatively low perceived hopefulness in the face of ecological crises. This aligns with Ojala's (2012) conceptualization of two distinct forms of eco‐hope: denial‐based hope, which is passive and linked to disengagement, and constructive hope, which is active and associated with greater environmental concern and action. In her research, constructive hope was positively associated with pro‐environmental behavior, while denial‐based hope had a negative influence. In line with this, Verplanken et al. (2020 study 3) found that eco‐determination (a related concept) was also positively associated with pro‐environmental behaviors. These findings suggest that understanding different forms and functions of eco‐hope may be critical to explaining not just emotional responses to climate change, but also the motivational dynamics behind individual and collective environmental engagement. From a coping perspective, such distinctions reflect differences between adaptive, meaning‐focused coping and more avoidant or disengaged responses (Folkman, 2008).

However, pathologizing eco‐emotions like eco‐anxiety would be doing a disservice to the lived experiences of individuals, as arguably eco‐anxiety is an appropriate response to current circumstances (Hogg et al., 2021; Pihkala, 2018). Addressing the climate and ecological crises requires large‐scale changes by government agencies. Focusing on individual changes can lead to meaning‐based individual‐coping (e.g. cutting meat from one's diet, using public transport or cycling, etc.) that may help individuals cope with eco‐emotions individually, but addressing the climate and ecological crises requires active, meaningful participation from government and corporate stakeholders and policy‐level changes at the community, national, and international levels.

### Eco‐anxiety vs eco‐fear

Anxiety as an emotion can be defined as “feeling of unease, such as worry or fear, that can be mild or severe” (NHS, 2022). Although similar to fear, anxiety is considered “future‐oriented, long‐acting response broadly focussing on a diffuse threat”; while fear is “appropriate, present‐oriented, and short‐lived response to a clearly identifiable and present threat” (APA, 2024). Studies among populations experiencing regular impacts of anthropogenic environmental degradation report high scores of eco‐fear due to facing consequences first‐hand (Galway & Field, 2023; Gibson et al., 2020; Hickman et al., 2021). Coupled with the lack of global, collaborative action in addressing the crises and the perception of their own lack of control over the situation, vulnerable populations will continue to experience debilitating eco‐emotions that will negatively impact their mental health. Despite contributing minimally towards global anthropogenic environmental degradation, it is low‐and‐middle‐income countries that are facing an overwhelming majority of the consequences (Gibson et al., 2020; IPCC, pg. 5, 2023; Pietromarchi, 2021; Suri, 2022; Simon et al., 2022).

### Meta‐analysis

Meta‐analytic results presented significant, positive moderate effect sizes in the relationship between eco‐anxiety and depression, anxiety, and stress, respectively. The relatively modest effect sizes reinforce that pathologizing eco‐anxiety would be unsuitable for many individuals and miss nuances behind climate and ecological crises (Pihkala, 2020; Verplanken & Roy, 2013). Some evidence, though, suggests that climate anxiety may vary according to values held, with this and other factors potentially predisposing some individuals towards greater climate anxiety (Kühner et al., 2025). The meta‐analytic findings are consistent with stress and coping theory, which suggests that chronic and uncontrollable stressors are associated with increased psychological distress (Lazarus & Folkman, 1984). The results may also be understood through appraisal‐based models, whereby the perceived severity and uncontrollability of ecological threats contribute to heightened emotional responses (Scherer, 2001).

A moderate effect size positive relationship was found between eco‐anxiety and general anxiety. Whilst some may argue that eco‐anxiety is just another manifestation of general anxiety, our results suggest a separation between these constructs, comparable with phenomena like test anxiety, which has also been reported to be distinct from general anxiety (Benson, 1989; Putwain et al., 2021). Individuals without general anxiety may nonetheless experience eco‐anxiety specific to lived experiences surrounding the climate and ecological crises, which could affect overall functioning and wellbeing (Cosh et al., 2024).

While low levels of stress (i.e. eustress) are beneficial as they motivate one to address issues faced, prolonged exposure to stressful situations or high levels of stress (i.e. distress) can incapacitate individuals, leading to worsening physical and mental health. In the context of eco‐emotions, modest levels of eco‐anxiety together with eco‐anger or (constructive) eco‐hope may motivate individuals to take action to address their concerns. However, high levels of eco‐anxiety, together with other eco‐emotions like eco‐fear or eco‐guilt, may incapacitate individuals and increase the risk of mental health difficulties. Understanding the relationship between levels of stress and eco‐anxiety, particularly in the context of eustress and distress, may play an important role in helping individuals develop adaptive coping strategies, such as pro‐environmental behaviors and climate activism, to help cope with negative eco‐emotions.

### Eco‐emotions vs climate‐emotions

Existing research on eco‐emotions has largely focused on affective responses to climate change, while comparatively little attention has been directed to broader ecological crises. Current measurement tools predominantly capture emotions specific to climate change, rather than encompassing emotional responses to the wider ecological crisis, including the systemic degradation of ecosystems through biodiversity loss, habitat destruction, species extinction, deforestation, and pollution (Maechler & Graz, 2024). A broader framing that captures emotional responses related to wider ecological events beyond climate change is therefore critical for a more comprehensive understanding of eco‐emotions within the context of contemporary environmental challenges.

### Defining eco‐anxiety

The review highlights the broad way in which eco‐anxiety has been defined, with different scales operationalizing the construct in different ways. While not an aim of the review, the heterogeneity in the operationalization of eco‐anxiety prompted further exploration, particularly given its prominence as the most frequently reported eco‐emotion. Some measures (e.g. Clayton & Karazsia, 2020; Stewart, 2021) place greater emphasis on cognitive dimensions such as worry or rumination, whereas others adopt broader or multidimensional approaches (e.g. Hogg et al., 2021). While these differences reflect ongoing efforts to capture a complex construct, they also contribute to heterogeneity in how eco‐anxiety is assessed across studies. The absence of a shared conceptual and operational definition of eco‐anxiety/climate‐anxiety has resulted in the use of diverse measurement approaches, contributing to variability in findings across the literature. This lack of consistency poses challenges for comparing results across studies and limits the reliability and generalizability of conclusions drawn from the existing evidence base. Establishing greater consensus in the definition and operationalization of eco‐anxiety/climate‐anxiety is needed to aid reliability and generalizability of results (Cosh et al., 2024; Kırımer‐Aydınlı et al., 2025; Ramsay et al., 2025).

### Limitations and strengths

Our results should be interpreted with caution, due to high heterogeneity among studies, reflecting in part differing aspects of the construct of eco‐anxiety being measured across scales. Moderator analyses did not yield statistically significant results, though the low number of studies in some groupings likely hindered the ability to appraise these differences. As further studies are published, such potential differences between groups could be further explored. Although a review of correlational data cannot be used to determine any causal relation, it supports the notion that mental health should be given greater consideration while discussing and implementing strategies related to the climate and ecological crises.

Most existing studies of eco‐emotions are based on high‐income nations, with few studies reporting levels of eco‐emotions from low‐and‐middle‐income countries and even fewer studies reporting relationships between eco‐anxiety and mental health outcomes in low‐and‐middle‐income countries. These low‐and‐middle‐income countries are often at greater risk from the climate and ecological crises, and more research on the effects of this on eco‐emotions and behavior in these nations is needed.

Another limitation is that, due to limited resources, the screening and inclusion of studies were conducted only by the first author. Finally, our review sought to explore the relationship between a range of eco‐emotions and mental health outcomes. Unfortunately, we were only able to conduct a meta‐analysis using eco‐anxiety, as insufficient studies had been conducted to date that explored other eco‐emotions.

To the best of our knowledge, this is the first meta‐analytic review to analyze the relationship between eco‐anxiety and depression, anxiety, and stress in young adults. The modest strength of the relationship between eco‐anxiety and anxiety supports the notion that eco‐anxiety is a separate construct. Our review also highlights observed levels of differing eco‐emotions and how these may differ across nations, perhaps relating to direct impacts of anthropogenic‐environmental degradation such as extreme weather events.

### Directions for future research

Primarily, a consensus needs to be reached regarding defining and operationalizing eco‐anxiety. Currently, the literature involves measurement tools that lead to high heterogeneity; homogeneity would add value by allowing generalizability and validity of results.

There is a need for greater exploration of eco‐emotions beyond eco‐anxiety, perhaps particularly in relation to eco‐anger and both adaptive and maladaptive aspects of eco‐hope. Exploring the relationship between eco‐anger and mental health could offer interesting findings that can help mental health workers and policy developers in resilience‐building, etc. Examining the activating or motivational aspect of eco‐anger in relation to individual and collective actions may provide insights for future strategies in climate and ecological crises communication and mitigation (Stanley et al., 2025). Studies focusing on low‐and‐middle‐income countries, particularly highly vulnerable populations, could help address aspects of climate and ecological justice, and enable greater understanding of their eco‐emotions, given the current focus of studies on the experience of individuals in the developed world. Similarly, there is a need to ensure validation of eco‐emotions scales in populations from low‐and‐middle‐income countries.

As highlighted by Kühner et al. (2025), examining the antecedents and consequences of climate anxiety within the framework of relevant predictors and moderators offers critical insights into the complex interplay between eco‐anxiety and related psychological, social, and environmental factors. Such an approach would enable a more nuanced understanding of how individual differences and contextual variables influence the manifestation and outcomes of climate‐related anxiety and associated eco‐emotional responses. Advancing this line of inquiry would be essential for informing both theoretical models of eco‐emotional processes and the development of targeted interventions that foster psychological resilience in the face of environmental change.

Finally, investigating eco‐emotions within specific cultural contexts (particularly those beyond Western, Eurocentric frameworks) would offer valuable insights into how these emotions are experienced and expressed across diverse populations. This is especially important for capturing lived‐experiences of indigenous communities, whose relationships with the environment are shaped by distinct cultural, historical, and ecological factors. Future research should prioritize culturally grounded methodologies to better understand and represent the plurality of eco‐emotional experiences worldwide (Cosh et al., 2024;Kühner et al., 2025).

## CONCLUSION

This study finds moderate to high levels of eco‐anxiety in nations that have been widely affected by natural disasters related to climate and ecological crises. Other developed nations, in which such natural disasters have had less effect or affect less of the nation, tended to report mild to moderate levels of eco‐anxiety. Studies found high levels of eco‐fear and eco‐sadness, with some of these eco‐emotions perhaps explained by moderate to high levels of helplessness, hopelessness, and powerlessness.

Our meta‐analyses showed significant, positive moderate correlations between eco‐anxiety and each of depression, anxiety and stress, supportive of the idea that eco‐emotions and emotions are related but separate constructs. There is a particular need for more research in this area with populations from low‐and‐middle‐income countries; and a need to evaluate the prevalence and effect of eco‐emotions beyond eco‐anxiety, particularly eco‐anger and potentially the role of positive (determined) and negative (denial) eco‐hope in affecting emotions and wellbeing.

Overall, these findings support a view of eco‐emotions as responses shaped by cognitive appraisal and coping processes, with both adaptive and maladaptive implications (Lazarus & Folkman, 1984; Scherer, 2001).

## CONFLICT OF INTEREST STATEMENT

The authors declared no potential conflicts of interest with respect to the research, authorship, and/or publication of this article.

## ETHICS STATEMENT

Not applicable.

## Supporting information


**Appendix A1:** Studies included for review (Table A.1.1.).
**Appendix A2:** Characteristics of included studies (Figures A.2.1‐A.2.2.).
**Appendix A3:** Traffic plots and summary plots of RQ1 and RQ2 (Figures A.3.1‐A.3.4.).
**Appendix A4:** Eco‐emotions scales and Average and weighted mean of eco‐anxiety reported by all studies included in review (Tables A.4.1.‐A.4.2.).
**Appendix A5:** Revised meta‐analytic results excluding ad‐hoc measurement scales.
**Appendix A6:** Baujat plots of meta‐analytic estimates to identify influential outliers (Figures A.6.1.‐A.6.3.).
**Appendix A7:** Results of Meta‐regression (moderator analyses) (Tables A.7.1.‐A.7.3.).
**Appendix A8:** Funnel plots of meta‐analytic estimates (Figures A.8.1.‐A.8.4.).

## Data Availability

The data that support the findings of this study are available from the corresponding author upon reasonable request.
