# Supplementary material for: A systematic review and meta‐analysis on the relationship of eco‐emotions on the mental health and wellbeing of young adults
Source: Appl Psychol Health Well Being. 2026 May 6;18:e70157. doi: 10.1111/aphw.70157 (PMC13147318; doi:10.1111/aphw.70157)
Supplement: Supplementary file 1 — Appendix A1: Studies included for review (Table A.1.1.). Appendix A2: Characteristics of included studies (Figures A.2.1‐A.2.2.). Appendix A3: Traffic plots and summary plots of RQ1 and RQ2 (Figures A.3.1‐A.3.4.). Appendix A4: Eco‐emotions scales and Average and weighted mean of eco‐anxiety reported by all studies included in review (Tables A.4.1.‐A.4.2.). Appendix A5: Revised meta‐analytic results excluding ad‐hoc measurement scales. Appendix A6: Baujat plots of meta‐analytic estimates to identify influential outliers (Figures A.6.1.‐A.6.3.). Appendix A7: Results of Meta‐regression (moderator analyses) (Tables A.7.1.‐A.7.3.). Appendix A8: Funnel plots of meta‐analytic estimates (Figures A.8.1.‐A.8.4.). [file APHW-18-0-s001.docx]

**Appendix**

[Appendix A1](#AppendixA1): Studies included for review (Table A.1.1.)

[Appendix A2](#AppendixA2): Characteristics of included studies (Figures A.2.1-A.2.2.)

[Appendix A3](#AppendixA3): Traffic plots and summary plots of RQ1 and RQ2 (Figures A.3.1-A.3.4.)

[Appendix A4](#AppendixA4): Eco-emotions scales and Average and weighted mean of eco-anxiety reported by all studies included in review (Tables A.4.1.-A.4.2.)

[Appendix A5](#AppendixA5): Revised meta-analytic results excluding ad-hoc measurement scales

[Appendix A6](#AppendixA6): Baujat plots of meta-analytic estimates to identify influential outliers (Figures A.6.1.-A.6.3.)

[Appendix A7](#AppendixA7): Results of Meta-regression (moderator analyses) (Tables A.7.1.-A.7.3.)

[Appendix A8](#AppendixA8): Funnel plots of meta-analytic estimates (Figures A.8.1.-A.8.4.)

**Appendix A1**

**Table A.1.1.** Studies included for review.

| **Author(s), year**  **Country** | **N for current review** | **Study Design** | **% Female sex** | **Population type** | **Eco-emotion Measure** | **Range and Mean (SD) of scores on eco-emotion measure** | **Mental Health Measure** | **Included in Meta-analysis** |
| --- | --- | --- | --- | --- | --- | --- | --- | --- |
| Abou Jaoude et al., 2024^+^  Lebanon | 763 | Cross-sectional survey | 63.4% | General population | CAS | [range 1-5]: **1.96** | Death Anxiety Scale | Yes |
| Clayton et al., 2020 study 1  USA | 197 | Cross-sectional survey | 40.61% | General population | CAS | [range 1-5]: **1.75 (.97)** | PHQ-4 | Yes |
| Clayton et al., 2020 study 2  USA | 199 | Cross-sectional survey | 32.16% | General population | CAS | [range 1-5]: **1.75 (1.07)** | PHQ-4 | Yes |
| Contreras et al., 2023  Belgium | 51 | Cross-sectional survey | N/A | General population | CAS | [range 1-5]: **2.13 (0.65)** | DASS_D  DASS_A  DASS_S | Yes |
| Daeninck et al., 2023^+^  UK | 473 | Cross-sectional survey | 53.7% | University students | CAS | [range 1-5]: **1.87** | N/A | No |
| Ediz et al., 2023  Turkey | 306 | Cross-sectional survey | 75.2% | University students | CAS | [range 1-5]: **2.20 (0.78)** | Beck Hopelessness Scale | Yes |
| Fekih-Romdhane et al., 2024^+^  Lebanon | 596 | Cross-sectional survey | 63.9% | General population | CAS | [range 1-5]: **1.98** | Scale of Death Anxiety | Yes |
| Hajek et al., 2023  Germany | 566 | Cross-sectional survey | 79.0% | General population | CAS (German adapted version) | [range 1-7]: **2.4 (1.3**) | GAD-7 | Yes |
| Heeren et al., 2022  Europe & Africa | 78 | Cross-sectional survey | 50.0% | General population | CAS | [range 1-5]: **2.30 (0.89)** | N/A | No |
| Larionow et al., 2022^+^  Poland | 106 | Cross-sectional survey | 57.05% | General population | CAS | [range 1-5]: **1.60** | PHQ | Yes |
| Lutz, 2023 study 5  Canada | 308 | Cross-sectional survey | 78.6% | University students | CAS | [range 1-5]: **1.73 (0.73)** | DASS_A;  DASS_D;  DASS_S | Yes |
| McBride, 2022  New Zealand | 260 | Cross-sectional survey | 84.23% | University students | CAS;  CCDS | [range 1-5]: **1.65 (0.66)**  Climate Hopelessness [range 1-4]: **2.16 (0.96)** | DASS_D  DASS_A | Yes |
| Nadarajah et al., 2022  France | 369 | Cross-sectional survey | 73.4% | General population | CAS | [range 1-7]: **2.06 (0.96)** | N/A | No |
| Niskanen et al., 2022^+^  Finland | 795 | Cross-sectional survey | 60.08% | General population | CAS | [range 1-5]: **1.53** | STAI-6 | Yes |
| Papadopoulou, 2021^+^  Greece, Europe, Canada | 98 | Cross-sectional survey | 68.37% | General population | CAS | [range 1-5]: **1.76** | GAD-7 | Yes |
| Pathak et al., 2024^+^  India | 190 | Cross-sectional survey | N/A | General population | CAS | [range 1-5]: **2.47** | WHOQOL-Brief (Psych) | Yes |
| Plohl et al., 2023  Slovenia | 442 | Cross-sectional survey | 75.8% | General population | CAS | [range 1-5]: **1.70 (0.72)** | DASS_A  DASS_S | Yes |
| Ramírez-López et al., 2023  Mexico | 468 | Cross-sectional survey | 65.38% | University students | CAS | [range 0-4]: **1.03 (1.04)** | GAD-7 | Yes |
| Reyes et al., 2021  Philippines | 433 | Cross-sectional survey | 66.5% | General population | CAS | [range 1-5]: **2.38 (.77)** | MHI-38 | Yes |
| Schwartz et al., 2022  USA | 284 | mixed method: cross-sectional survey; semi-structured interview | 78.9% | University students | CAS | [range 1-5]: **1.41 (0.54)** | GAD-7;  PHQ-8 | Yes |
| Simon et al., 2022^+^  Philippines | 452 | Cross-sectional survey | N/A | University students | CAS | [range 1-5] **2.33** | N/A | No |
| Tam et al., 2023  China, India, Japan, USA | 575 | Cross-sectional survey | China: 49.0%  India: 49.0%  Japan: 52.4%  USA: 51.7% | General population | CAS | [range 1-5]:  China: **1.97 (0.63)**  India: **2.65 (0.8**)  Japan: **1.5 (0.63)**  USA: **2.02 (0.92)** | N/A | No |
| Whitmarsh et al., 2022  UK | 891 | Cross-sectional survey | 53.0% | General population | CAS | [range 1-5]: **1.25 (0.46)** | GAD-7 | Yes |
| Wullenkord et al., 2021  Germany | 212 | Cross-sectional survey | 51.14% | General population | CAS | [range 1-7]: **1.94 (0.94)** | PHQ-4 | Yes |
| Çimşir et al., 2024^+^  Turkey | 185 | Cross-sectional survey | 61.08% | University students | HEAS | [range 0-3]: **0.97** | Brief Symptom Inventory (BSI) | Yes |
| Er et al., 2024  Turkey^+^ | 609 | Cross-sectional survey | 84.2% | University students | HEAS | [range: 0-3]: **2.04** | DASS_D  DASS_A  DASS_S | No |
| Heinzel et al., 2023  Germany | 134 | Cross-sectional survey | N/A | University students | HEAS | [range 0-3]: **0.80 (0.65)** | DASS_D  DASS_A  DASS_S | Yes |
| Hogg et al., 2021 study 1  Australia | 296 | Cross-sectional survey | 57.77% | University students | HEAS | [range 0-3]: **0.43 (0.57)** | GAD-7 | Yes |
| Hogg et al., 2021 study 2  New Zealand | 715 | Longitudinal survey | 74.55% | University students | HEAS | [range 0-3]: **0.73 (0.83)** | DASS-21 | Yes |
| Hogg et al., 2024 study 1  UK | 76 | Cross-sectional survey | 53.95% | General population | HEAS | [range 0-3]: **1.27 (0.39)** | PSWQ | Yes |
| Hogg et al., 2024 study 2  USA | 192 | Cross-sectional survey | 54.17% | General population | HEAS | [range 0-3]: **1.52 (0.60)** | PSWQ | Yes |
| Lutz, 2023b  Canada | 132 | Cross-sectional survey | 72.6% | University students | HEAS (adapted version) | [range 1-5]: **2.13 (0.82)** | Positive and Negative Affect Schedules | Yes |
| Rodríguez Quiroga et al., 2024  Spain | 548 | Cross-sectional survey | 86.0% | University students | HEAS | [range 0-3]: **0.92 (0.65)** | N/A | No |
| Sampaio et al., 2023  Portugal | 623 | Cross-sectional survey | 81.5% | University students | HEAS  (Portuguese adapted version) | [range 0-3]: **2.86**  Standard deviation not provided | N/A | No |
| Türkarslan et al., 2023 study 1  Turkey | 605 | Cross-sectional survey | 69.92% | General population | HEAS | [range 0-3]: **0.83 (0.68)** | DASS_A  DASS_D  DASS_S | Yes |
| Vercammen et al., 2023^+^  USA | 2834 | Cross-sectional survey | 49.8% | General population | HEAS (adapted version) | [range 0-3]: **1.00** | N/A | No |
| Lutz, 2023 study 1  Canada | 238 | Cross-sectional survey | N/A | University students | Helm et al., 2018 ecological stress scale | [range 1-5]: **3.35 (0.97)** | N/A | No |
| Lutz, 2023 study 2  Canada | 443 | Cross-sectional survey | 77.7% | University students | Helm et al., 2018 ecological stress scale | [range 1-5]: **3.48 (1.02)** | DASS_A;  DASS_D;  DASS_S | Yes |
| Lutz, 2023 study 3  China | 1635 | Cross-sectional survey | 53.0% | University students | Helm et al., 2018 ecological stress scale | [range 1-5]: **3.38 (0.79)** | DASS_A;  DASS_D;  DASS_S  (Chinese adapted version) | Yes |
| Kulcar et al., 2022  Austria | 314 | Cross-sectional survey | 73.2% | University students | CCDS | [range 1-4]:    ClimateChange Anxiety: **2.17 (1.02)**  ClimateChange Hopelessness: **2.46 (0.99)** | N/A | No |
| Searle et al., 2010  Australia | 275 | Cross-sectional survey | 61.0% | General population | CCDS | [range 0-4]:  ClimateChangeAnxiety: **1.10**^+^  ClimateChangeHopelessness: **2.64 (2.25)** | DASS-21 | Yes |
| Stewart, 2021 study 3  USA^+^ | 417 | Cross-sectional survey (S3) | 85.0% | University students | CCWS | [range 1-5]: **2.44** | DASS | Yes |
| Galway et al., 2023  Canada | 1000 | Cross-sectional survey | 54.0% | General population | Ad-hoc questionnaire | [range 1-7]: **4.13 (2.60)** | N/A | No |
| Gibson et al., 2020  Tuvalu | 31 | Cross-sectional survey | 41.94% | General population | Ad-hoc questionnaire | [range 1-4]: **2.04 (0.68)** | N/A | No |
| Hickman et al., 2021  Global | 10000 | Cross-sectional survey | 48.6% | General population | Ad-hoc questionnaire | Worried about climate change [range 1-5]: **3.7 (1.7)**  Feelings of betrayal by governments [range 1-5]: **2.7 (1.0)** | N/A | No |
| Lawrance et al., 2022^+^  UK | 530 | Cross-sectional survey | 63.0% | General population | Ad-hoc questionnaire | [range 0-4]: **1.64** | GAD-7; PHQ-9; | No |
| Leviston et al., 2021 (U.D^*^)  Australia | 1214 | Cross-sectional survey | 68.0% | General population | Ad-hoc questionnaires | EcoAnxiety [range 0-100]: **56.92 (27.76)**  EcoAnger [range 0-10]: **56.48 (29.27)**  EcoDepressed [range 0-100]: **40.12 (28.06)** | DASS_A  DASS_D | Yes |
| Maran et al., 2021^+^  Italy | 312 | Cross-sectional survey | 74.0% | University students | Ad-hoc questionnaire: Modified version of STAI | [range 1-5]: **3.41** | N/A | No |
| Ogunbode et al., 2021  Global | 10143 | Cross-sectional survey | 63.4% | General population | Ad-hoc questionnaire: Modified version of STAI | [range 1-7]: **3.23 (0.83)** | 1-item from Ontario Health Survey | No |
| Ogunbode et al., 2022  Global | 12246 | Cross-sectional survey | 63.9% | General population | Ad-hoc questionnaire: Modified version of STAI based on state Anxiety component. | [range 1-5]: **3.21 (.82)** | N/A | No |
| Prencipe et al., 2023  Tanzania | 2053 | Cross-sectional survey | 45.0% | General population | Ad-hoc 1-item for climate distress | [range 1-4]: **1.68 (0.86)** | Centre for Epidemiological Studies Depression Scale (CES-D10) | Yes |
| Sciberras et al., 2022  Australia | 3037 | Longitudinal study (Wave 8) | 49.78% | General population | Ad-hoc 1-item questionnaire | [range 1-5]: **2.51 (1.47)** | N/A | No |
| Stanley et al., 2021  Australia | 1045 | Cross-sectional survey | 72.6% | General population | Ad-hoc questionnaires | EcoAnger [range 0-100]: **53.35 (29.75)**  EcoAnxiety [range 0-100]: **52.62 (28.93)**  EcoDepressed [range 0-100]: **37.37 (27.65)** | DASS_A  DASS_D  DASS_S | Yes |
| Swim et al., 2022  USA | 4888 | Longitudinal survey | N/A | General population | Ad-hoc questionnaire | Worry [range: 0-3]: **1.37(0.02 SE)**  Anger [range 0-3]: **1.23 (0.04 SE)**  Guilt [range 0-3]: **0.96**  **(0.03 SE)** | N/A | No |
| Verplanken et al., 2013  USA & Europe | 132 | Cross-sectional survey | 59.09% | University students | Ad-hoc questionnaire: Habitual ecological worrying | [range 1-5]: **3.24 (0.83)** | PSWQ | No |
| Verplanken et al., 2020 study 1  USA & Europe | 266 | Cross-sectional survey | 72.93% | General population | Ad-hoc questionnaire: HINT_GW | [range 1-5]: **2.85 (.07)** | PSWQ | Yes |
| Verplanken et al., 2020 study 2  USA & Europe | 293 | Cross-sectional survey | 73.04% | General population | Ad-hoc questionnaire: HINT_GW | [range 1-5]: **2.88 (0.89)** | PSWQ | Yes |
| Verplanken et al., 2020 study 3  USA, UK, Europe | 306 | Cross-sectional survey | 67.97% | General population | Ad-hoc questionnaire: HINT_GW | [range 1-5]: **3.11 (0.72)** | PNAS | Yes |
| Yatirajula et al., 2023^+^  India | 600 | Cross-sectional survey | 51.7% | General population | Ad-hoc scale measuring climate change concern | [range: 0-3]: **1.23** | N/A | No |

**Note:** UD= Unpublished Data; CAS= Climate-anxiety scale; HEAS= Hogg eco-anxiety scale; CCDS= Climate change distress scale; STAI= State-trait anxiety inventory; CCWS= Climate change worry scale; HINT_GW= Habit index of negative thinking_Global warming; PHQ= Patient health questionnaire; GAD= Generalised anxiety disorder; DASS= Depression, anxiety, stress scale; PSWQ= Penn state worry questionnaire; PSS= Perceived stress scale; MHI= Mental health inventory; PNAS= Positive & negative affect schedule; WHO= World health organisation. ^+^ = Means and SDs were reported as total scores in these studies and recalculated means have been presented accordingly; SDs were not available.

**Appendix A2**

Characteristics of included studies:

**Figure A.2.1.** Geographic distribution of studies per country

**Figure A.2.2.** Publication of studies over time.

**Figure A.2.1.** Geographic distribution of studies per country

**Figure A.2.2.** Publication of studies over time. **Note:** The year 2024 is until November 2024 only.

**Appendix A3**

Risk of bias (RoB) traffic plots and summary bar plots for review question 1 and review question 2:

**Figure A.3.1.** RoB traffic plot for studies addressing review question 1

**Figure A.3.2.** The weighted (by N) summary bar plot of risk of bias (RoB) judgement for review question 1

**Figure A.3.3.** RoB traffic plot for studies addressing review question 2

**Figure A.3.4.** The weighted (by N) summary bar plot of risk of bias (RoB) judgement for Eco-anxiety and Mental Health & Wellbeing relationship (i.e. review question 2)


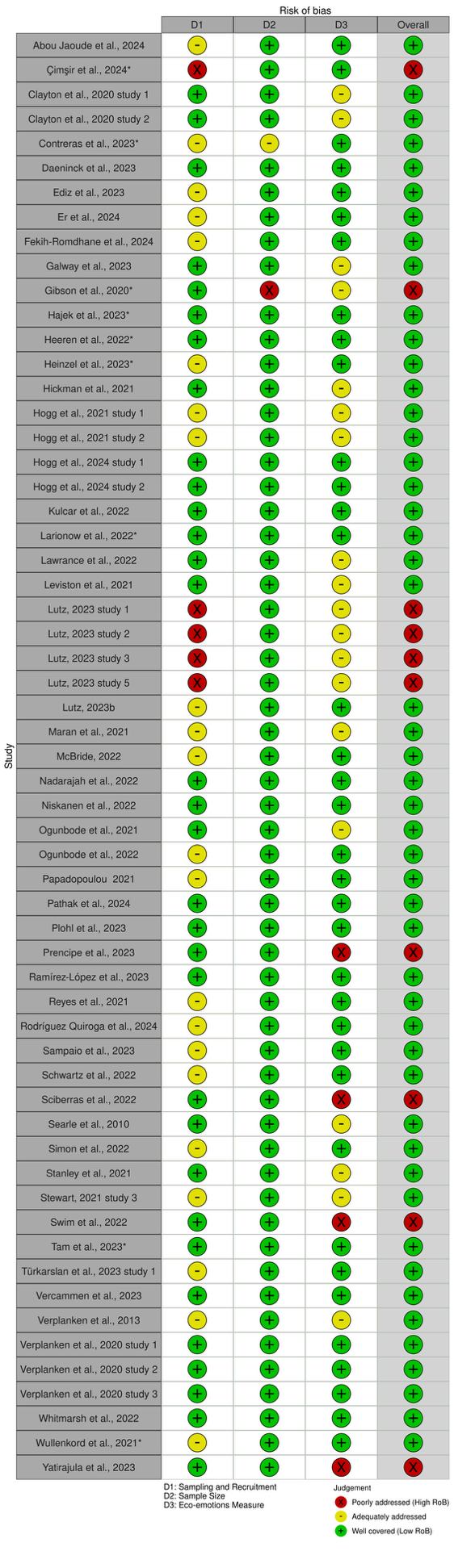


**Figure A.3.1.** RoB traffic plot for studies addressing review question 1


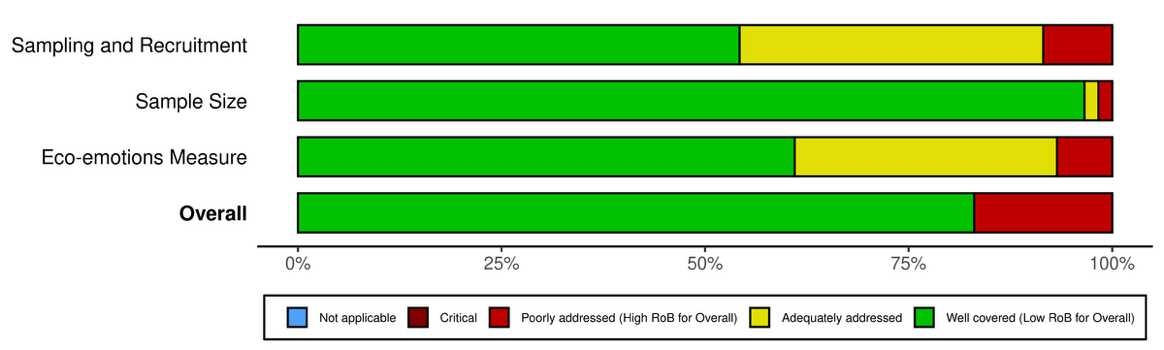


**Figure A.3.2.** The weighted (by N) summary bar plot of risk of bias (RoB) judgement for levels of climate and ecological emotions (i.e. review question 1)


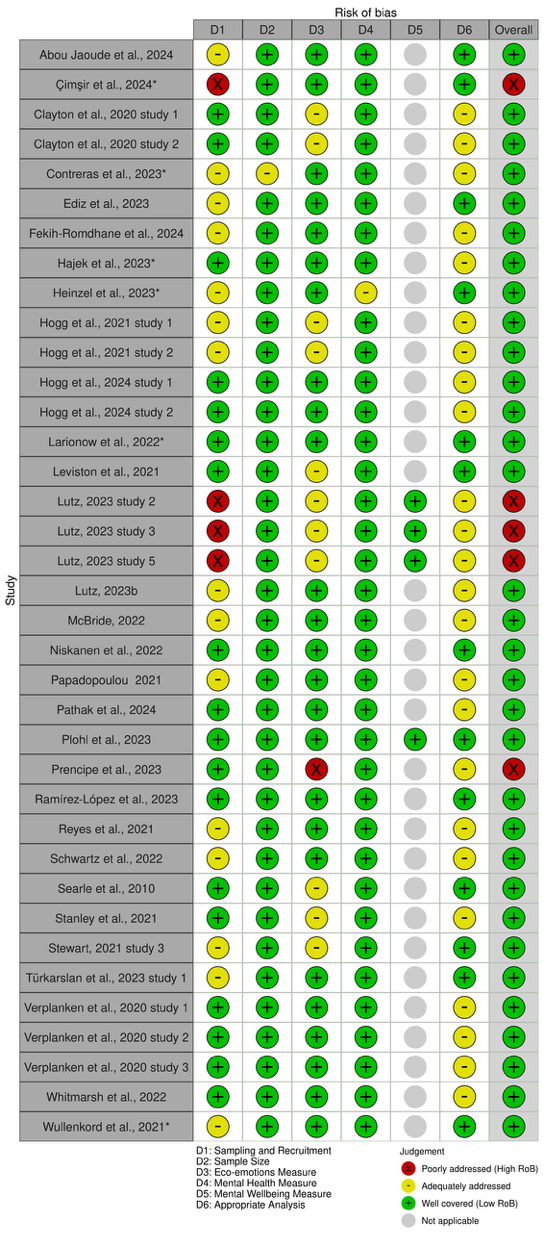


**Figure A.3.3.** RoB traffic plot for studies addressing review question 2

**
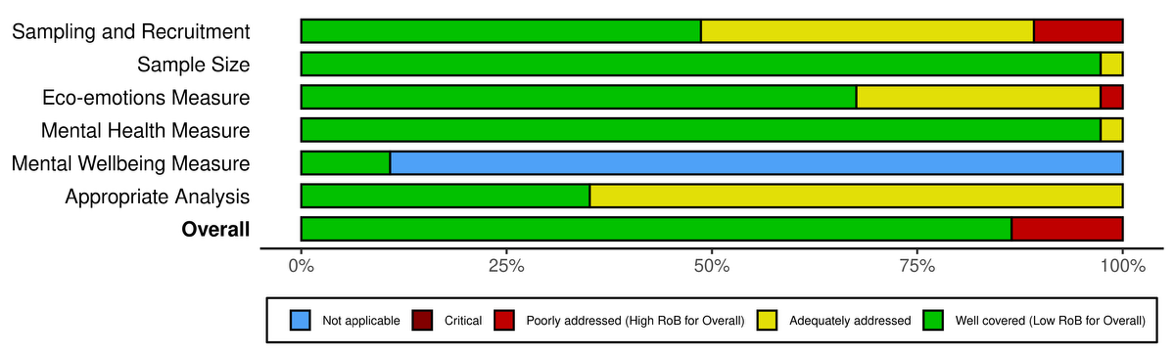
**

**Figure A.3.4.** The weighted (by N) summary bar plot of risk of bias (RoB) judgement for Eco-anxiety and Mental Health & Wellbeing relationship (i.e. review question 2)

**Appendix A4**

Eco-emotions scale, and Average and weighted mean of eco-anxiety by all studies included in review:

**Table A.4.1.** Eco-emotions scales

**Table A.4.2.** Average mean of eco-anxiety reported by all studies included in review.

**Table A.4.1.** Eco-emotions scales**.**

| **Name of scale** | **Number of items** | **Range** | **Subscales** | **Type of scale** |
| --- | --- | --- | --- | --- |
| Eco-emotions  Searle & Gow, 2010 | 12 | 0-3 | 1. Climate change anxiety (nine items)* 2. Climate change hopelessness (three items) | Emotion clusters.  Frequency scale. |
| Eco-worry  Verplanken et al., 2020. | 12 | 1-5 | 1. Habitual worry about global warming* | Emotion clusters. Frequency scale. |
| Climate-anxiety scale (CAS)  Clayton & Karazsia, 2020. | 13 | 1-5 | 1. Cognitive-emotional impairment (eight items)* 2. Functional impairment (five items) | Aims to capture a construct known as 'a chronic fear of environmental doom'. Frequency scale. |
| Hogg Eco-anxiety Scale  (HEAS)  Hogg et al., 2021 | 13 | 0-3 | 1. Affective symptoms (four items)* 2. Behavioural symptoms (three items) 3. Rumination (three items) 4. Personal impact anxiety (three items) | Frequency scale. |
| Climate change worry scale  (CCWS)  Stewart, 2021 | 10 | 1-5 | 1. Climate change worry* | Aims to capture ‘a single construct of personal worry about climate change.’ Frequency scale |
| Eco-emotions  Stanley et al., 2021 | 03 | 1-100 | 1. Eco-depression* 2. Eco-anxiety* 3. Eco-anger* | Intensity scale. |
| Eco-anxiety ad-hoc scale  Maran and Begotti, 2021 | 07 | 7-35 | 1. Eco-anxiety* | Emotion clusters. Intensity scale. |
| Climate and eco-anxiety ad-hoc scale  Hickman et al., 2021 | 08 | 1-5  0-4 | 1. Climate and eco-anxiety* 2. Government responses to climate change* | Emotion clusters. Intensity scale. |
| Helm ecological stress scale  Lutz et al., 2023 study 1; study 2; study 3; study 5 | 04 | 1-5 | 1. Eco-anxiety* | Intensity scale. |

**Note:** * = subscale used for the review

**Table A.4.2.** Average mean of eco-anxiety reported by all studies included in review.

| **Scale name** | **Average mean** | **Weighted Mean (by *N*)** | **Range of scale** | **Number of studies** |
| --- | --- | --- | --- | --- |
| CAS | 1.91 | 1.84 | 1-5 | 23 |
| CAS (Modified version) | 2.13 | 2.21 | 1-7 | 3 |
| CAS (Mexican version) | 1.03 | 1.03 | 0-4 | 1 |
| HEAS | 1.22 | 1.20 | 0-3 | 11 |
| HEAS (adapted by Lutz et al., 2023b) | 2.13 | 2.13 | 1-5 | 1 |
| HINT_GW | 2.95 | 2.95 | 1-5 | 3 |
| Helm Ecological Stress Scale | 3.40 | 3.40 | 1-5 | 3 |
| CCDS | 1.64 | 1.67 | 0-4 | 2 |
| Ecoanxiety (developed by Stanley et al., 2020) | 55.14 | 55.27 | 0-100 | 2 |
| CCWS | 2.44 | 2.44 | 1-5 | 1 |
| Ad-hoc scale (Galway et al., 2023) | 4.13 | 4.13 | 1-7 | 1 |
| Ad-hoc scale (Gibson et al., 2020) | 2.04 | 2.04 | 1-4 | 1 |
| Ad-hoc scale (Hickman et al., 2021) | 3.7 | 3.7 | 1-5 | 1 |
| Ad-hoc scale (Lawrance et al., 2022) | 1.64 | 1.64 | 0-4 | 1 |
| Ad-hoc scale (Maran et al., 2021) | 3.41 | 3.41 | 1-5 | 1 |
| Ad-hoc scale (Ogunbode et al., 2021) | 3.23 | 3.23 | 1-7 | 1 |
| Ad-hoc scale (Ogunbode et al., 2022) | 3.21 | 3.21 | 1-5 | 1 |
| Ad-hoc scale (Prencipe et al., 2023) | 1.68 | 1.68 | 1-4 | 1 |
| Ad-hoc scale (Sciberras et al., 2022) | 2.51 | 2.51 | 1-5 | 1 |
| Ad-hoc scale (Swim et al., 2022) | 1.37 | 1.37 | 0-3 | 1 |
| Ad-hoc scale (Verplanken et al., 2013) | 3.24 | 3.24 | 1-5 | 1 |
| Ad-hoc scale (Yatirajula et al., 2023) | 1.23 | 1.23 | 0-3 | 1 |

**Note:** CAS= climate-anxiety scale; HEAS= Hogg eco-anxiety scale; HINT_GW= habit index of negative thinking_Global warming; CCDS= climate change distress scale; CCWS= climate change

**Appendix A5**

Revised meta-analytic results excluding ad-hoc measurement scales

A separate meta-analysis was conducted of the relationship between eco-anxiety and depression, by removing studies that used ad-hoc scales. Leviston et al., 2021; Prencipe, 2023; and Stanley et al., 2021 were removed. The total number of studies was 18 and total number of participants was 6464. The effect size increased slightly, to *r* = 0.31, 95%CI [0.22; 0.39], *p* < .0001, *k* = 18, *o* = 6464.

A separate meta-analysis was conducted of the relationship between eco-anxiety and anxiety, by removing studies that used ad-hoc scales. Leviston et al., 2021; Stanley et al., 2021; and Verplanken et al., 2020 study 1; 2020 study 2; and 2020 study 3 were removed. The total number of studies was 28 and total number of participants was 11568. The effect size remained the same, *r* = 0.34, 95%CI [0.28; 0.40], *p* < .0001, *k* = 28, *o* = 11568.

A separate meta-analysis was conducted of the relationship between eco-anxiety and stress, by removing studies that used ad-hoc scales. Stanley et al., 2021 was removed. The remaining number of studies was nine and total number of participants was 4974. The effect size remained the same, *r* = 0.30, 95%CI [0.20; 0.39], *p* < .0001, *k* = 9, *o* = 4974.

**Appendix A6**

Baujat plots of meta-analytic estimates to identify influential outliers:

**Figure A.6.1** Baujat plot for meta-analysis of Eco-anxiety and Depression

**Figure A.6.2.** Baujat plot for meta-analysis of Eco-anxiety and Anxiety

**Figure A.6.3** Baujat plot for meta-analysis of Eco-anxiety and Stress


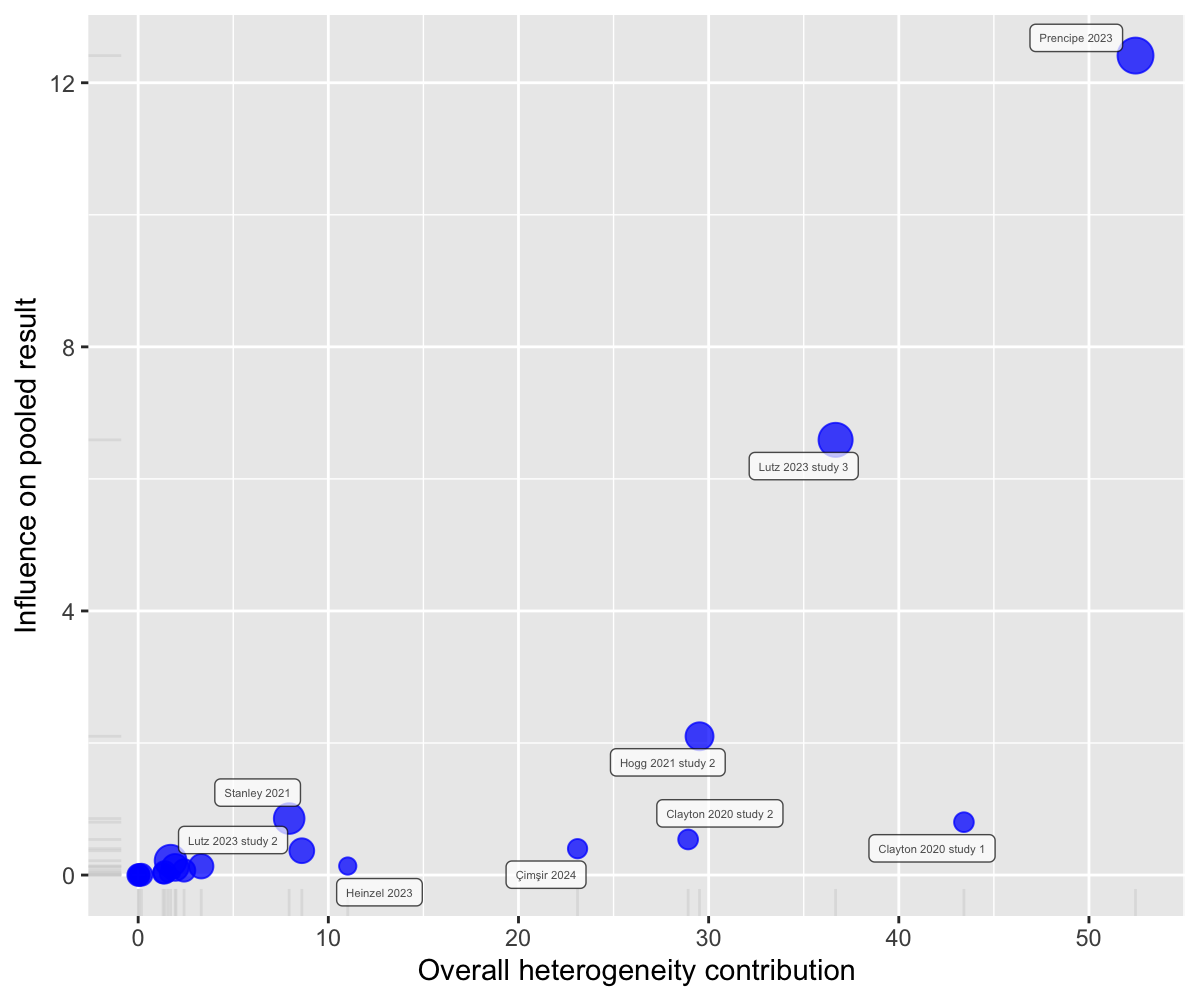


**Figure A.6.1.** Baujat plot for meta-analysis of Eco-anxiety and Depression


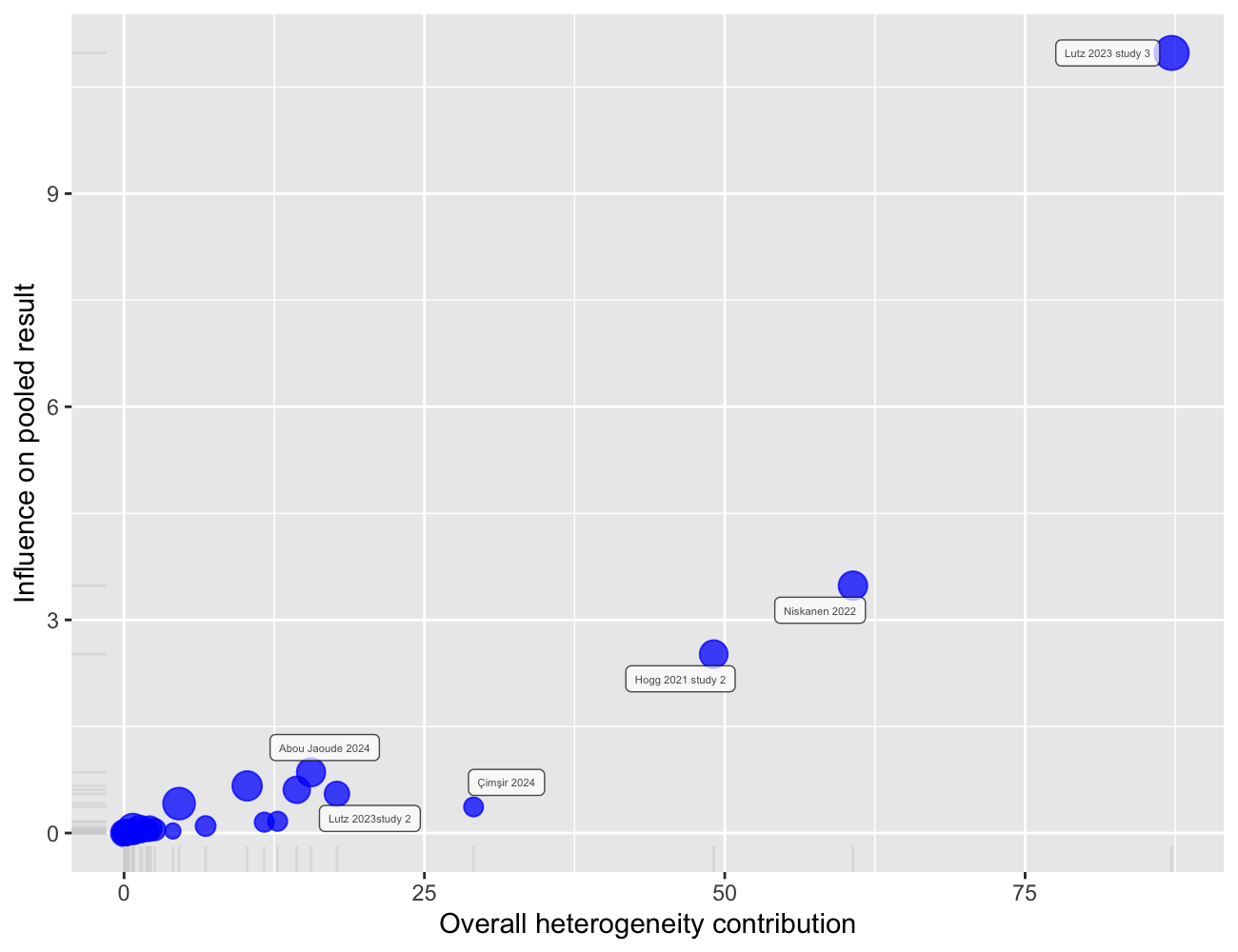


**Figure A.6.2.** Baujat plot for meta-analysis of Eco-anxiety and Anxiety


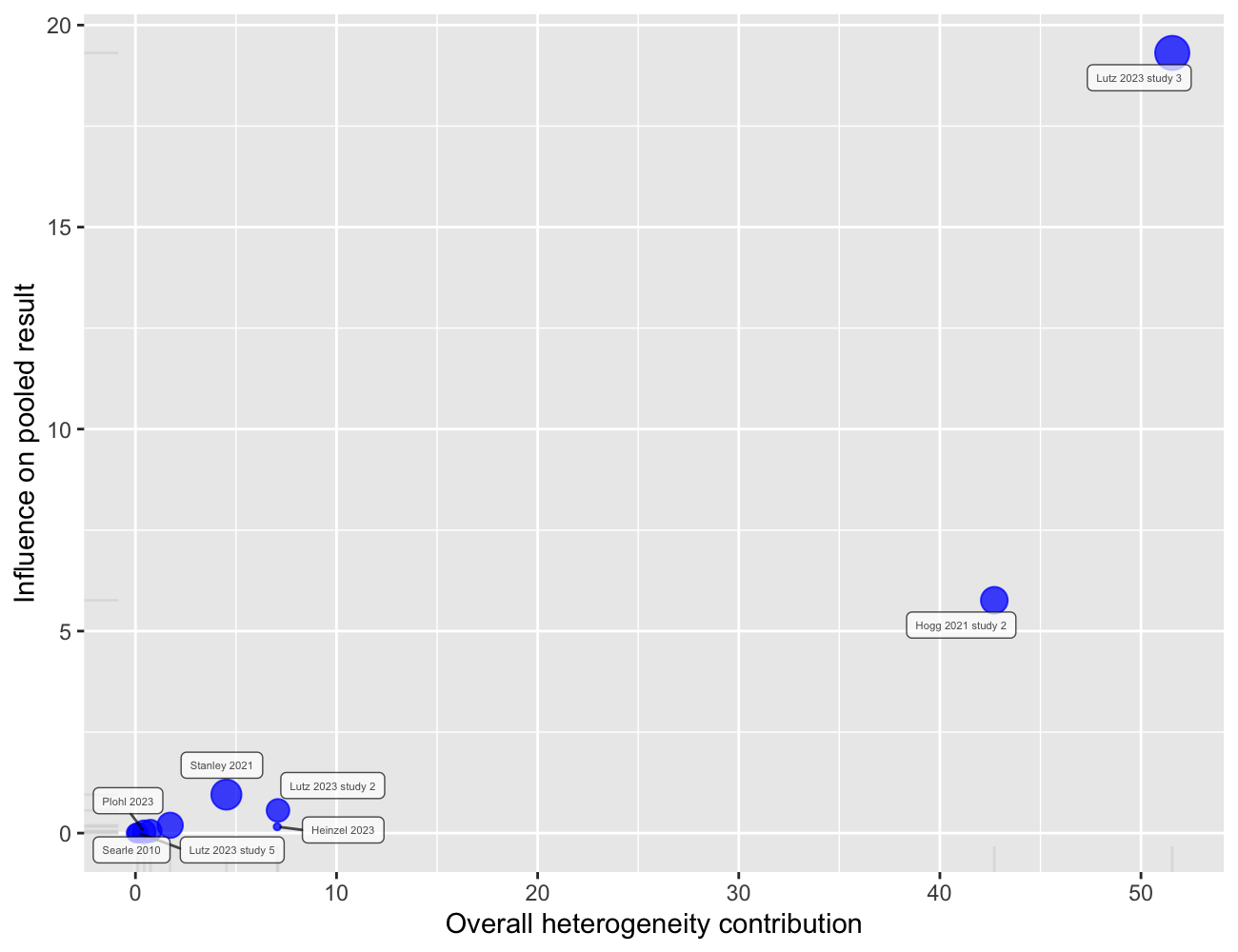


**Figure A.6.3.** Baujat plot for meta-analysis of Eco-anxiety and Stress

**Appendix A7**

**Table A.7.1.** Meta-regression analyses for Eco-anxiety and Depression

**Table A.7.2.** Meta-regression analyses for Eco-anxiety and Anxiety

**Table A.7.3.** Meta-regression analyses for Eco-anxiety and Stress

**Meta-regression (moderator analyses)**

The included studies were classified for their “economic status” based on the country of study using World Bank (2024), where countries were categorised as high-income, upper-middle income, lower-middle income, or low-income. “Eco-anxiety scale” was categorised based on climate-anxiety scale (Clayton and Karazsia, 2020) and Hogg eco-anxiety scale (Hogg et al., 2021). Two other categorical variables were based on “population”, and “risk of bias” of study. Continuous variables of “climate risk vulnerability”, “climate risk readiness”, and “ND-GAIN Index” were country-based scores calculated by ND-GAIN (2025) that calculated a country’s risk vulnerably on a scale of 1-0 with lower scores indicating lower risk; readiness on a scale of 1-0 with higher scores indicating higher readiness to dealing with climate-related challenges. A composite ND-GAIN index score was calculated with higher scores indicating a country’s better position in facing climate change related risk (ND-GAIN, 2025). Three other continuous variables were “percentage of female sex in sample”, “mean age”, and “percentage of sample with university education” that were weighted by *N* for the relationship between eco-anxiety and depression/anxiety. Categorical variable of “population” of study; continuous variables of “climate risk vulnerability” (ND-GAIN, 2025), “climate risk readiness” (ND-GAIN, 2025) “ND-GAIN Index” (ND-GAIN, 2025), “percentage of female sex in sample”, and “mean age” were used for the relationship between eco-anxiety and stress. Number of studies per category was guided by Fu et al., (2011). We limited analysis to subgroups with a minimum of four studies for categorical variables and less than ten studies for continuous variables.

No statistically significant moderator variables were found for the relationship between eco-anxiety and depression. Meta-regression outcomes and heterogeneity statistics for the eco-anxiety and depression analysis are shown in [Table A.7.1](#TableA71).

No statistically significant moderator variables were found for the relationship between eco-anxiety and anxiety. The direction of the estimate for climate risk readiness was negative, suggesting that the effect size gets weaker as the country is underprepared, reinforcing findings from the literature (IPCC, 2023). Meta-regression outcomes and heterogeneity statistics for the eco-anxiety and anxiety analysis are shown in [Table A.7.2](#TableA72).

No statistically significant moderator variables were found for the relationship between eco-anxiety and stress. Meta-regression outcomes and heterogeneity statistics for the eco-anxiety and stress analysis are shown in [Table A.7.3](#TableA73).

A lack of statistically significant results in the meta-regression analyses could be explained due to the low number of studies, and the high heterogeneity among the studies. High heterogeneity might suggest that the moderator variables are not the only factor influencing the variation in effect sizes, and further exploration of potential moderators could be needed. Additionally, due to the low number of studies in the literature, the regression-analyses may be updated once more studies are published.

**Table A.7.1.** Meta-regression analyses for Eco-anxiety and Depression. Note: k: Number of studies. SE: Standard error. p: Significance value. CI: Confidence intervals. CAS: Climate-anxiety scale. HEAS: Hogg eco-anxiety scale. R^2^: Proportion of variance explained.

| **Moderators** | ***k*** | **Estimate** | **SE** | **t** | ***p*** | **95% CI** |
| --- | --- | --- | --- | --- | --- | --- |
| **Population**  Intercept (Reference: General population)  University students  Test of moderators  Heterogeneity | 21  *F*(1, 19) = 0.36, *p* = 0.56. *R^2^* = 0.00%  *Q* = 257.42, *p* < .0001. τ2 = 0.03. *I^2^* = 92.89% | 0.32  -0.05 | 0.06  0.08 | 5.62  -0.60 | < .0001  < .56 | 0.20; 0.44  -0.21; 0.12 |
| **Climate risk vulnerability**  Intercept  Climate risk vulnerability  Test of moderators  Heterogeneity | 21  *F*(1, 19) = 2.03, *p* = 0.17. *R^2^* = 6.75%  *Q* = 176.37, *p* < .0001. τ2 = 0.03. *I^2^* = 91.54% | 0.65  -1.09 | 0.25  0.77 | 2.60  -1.43 | < .02  < .17 | 0.13; 1.18  -2.70, 0.51 |
| **Climate risk readiness**  Intercept  Climate risk readiness  Test of moderators  Heterogeneity | 21  *F*(1, 19) = 1.33, *p* = 0.26. *R^2^* = 3.14%  *Q* = 174.81, *p* < .0001. τ2 = 0.03. *I^2^* = 91.91% | 0.03  0.43 | 0.23  0.37 | 0.14  1.16 | < .89  < .26 | -0.45; 0.52  -0.35; 1.22 |
| **Climate risk ND-GAIN Index**  Intercept  Climate risk ND-GAIN Index  Test of moderators  Heterogeneity | 21  *F*(1,19) = 1.66, *p* = 0.21. *R^2^* = 4.93%  *Q* = 173.44, *p* < .0001. τ2 = 0.03. *I^2^* = 91.72% | -0.13  0.01 | 0.34  0.01 | -0.39  1.29 | < .70  < .21 | -0.84; 0.57  -0.00; 0.02 |
| **Eco-anxiety scale**  Intercept (Reference: CAS)  HEAS  Test of moderators  Heterogeneity | 14  *F*(1, 12) = 0.3700, *p* = 0.55. *R^2^* = 0.00%  *Q* = 84.83, *p* < .0001. . τ2 = 0.03. *I^2^* = 87.25% | 0.34  0.06 | 0.06  0.10 | 5.51  0.61 | < .00  < .55 | 0.20; 0.47  -0.16; 0.28 |
| **Economic status**  Intercept  Upper-middle  Test of moderators  Heterogeneity | 20  *F*(1,18) = 0.58, *p* = 0.46. *R^2^* = 0.00%  *Q* = 151.96, *p* < .0001. τ2 = 0.03. *I^2^* = 90.86% | 0.33  -0.07 | 0.04  0.10 | 7.42  -0.76 | < .0001  < .46 | 0.23; 0.42  -0.27; 0.13 |
| **Sex**  Intercept  Female %  Test of moderators  Heterogeneity | 18  *F*(1, 16) = 1.67, *p* = 0.22. *R^2^* = 1.59%  *Q* = 217.26, *p* < .0001. τ2 = 0.03. *I^2^* = 93.66% | 0.52  -0.00 | 0.19  0.00 | 2.75  -1.29 | < .01  < .22 | 0.12; 0.92  -0.01; 0.00 |
| **Mean age**  Intercept  Age  Test of moderators  Heterogeneity | 13  *F*(1, 11) = 0.09, *p* = 0.76. *R^2^* = 0.00%  *Q* = 113.98, *p* < .0001. τ2 = 0.02. *I^2^* = 89.96% | 0.40  -0.01 | 0.41  0.02 | 0.95  -0.31 | < .36  < .76 | -0.52; 1.31  -0.35; 0.04 |
| **University education**  Intercept  University education %  Test of moderators  Heterogeneity | 16  *F*(1, 14) = 0.12, *p* = 0.74. *R^2^* = 0.00%  *Q* = 144.19, *p* < .0001. τ2 = 0.03. *I^2^* = 89.95% | 0.35  -0.00 | 0.17  0.00 | 2.04  -0.34 | < .06  < .74 | -0.02; 0.71  -0.00; 0.00 |
| **Risk of bias**  Intercept (Reference: High risk of bias)  Low risk of bias  Test of moderators  Heterogeneity | 21  *F*(1, 19) = 2.13, *p* = 0.16. *R^2^* = 7.96%  Q = 142.92, *p* < .0001. τ2 = 0.03. *I^2^* = 91.88% | 0.20  0.13 | 0.07  0.09 | 2.71  1.46 | < .01  < .16 | 0.05; 0.36  -0.06; 0.31 |

**Table A.7.2.** Meta-regression analyses for Eco-anxiety and Anxiety. Note: k: Number of studies. SE: Standard error. p: Significance value. CI: Confidence intervals. CAS: Climate-anxiety scale. HEAS: Hogg eco-anxiety scale. R^2^: Proportion of variance explained.

| **Moderators** | ***k*** | **Estimate** | **SE** | **t** | ***p*** | **95% CI** |
| --- | --- | --- | --- | --- | --- | --- |
| **Population**  Intercept (Reference: general population )  University students  Test of moderators  Heterogeneity | 33  *F*(1, 31) = 0.11, *p* = 0.74. *R^2^* = 0.00%  *Q* = 330.80, *p* < .0001. τ2 = 0.02. *I^2^* = 89.61% | 0.34  0.02 | 0.03  0.06 | 10.16  0.33 | < .0001  < .74 | 0.26; 0.41  -0.10; 0.13 |
| **Climate risk vulnerability**  Intercept  Climate risk vulnerability  Test of moderators  Heterogeneity | 29  *F*(1, 27) = 0.30, *p* = 0.59. *R^2^* = 0.00%  *Q* = 336.47, *p* < .0001. τ2 = 0.02. *I^2^* = 91.36% | 0.23  0.36 | 0.21  0.65 | 1.09  0.55 | < .28  < .59 | -0.20; 0.67  -0.97, 1.68 |
| **Climate risk readiness**  Intercept  Climate risk readiness  Test of moderators  Heterogeneity | 29  *F*(1, 27) = 0.28, *p* = 0.60. *R^2^* = 0.00%  *Q* = 337.03, *p* < .0001. τ2 = 0.02. *I^2^* = 91.33% | 0.42  -0.12 | 0.14  0.22 | 3.09  -0.53 | < .00  < .60 | 0.14; 0.70  -0.58; 0.34 |
| **Climate risk ND-GAIN Index**  Intercept  Climate risk ND-GAIN Index  Test of moderators  Heterogeneity | 29  *F*(1,27) = 0.30, *p* = 0.59. *R^2^* = 0.00%  *Q* = 336.76, *p* < .0001. τ2 = 0.02. *I^2^* = 91.33% | 0.47  -0.00 | 0.22  0.00 | 2.15  -0.55 | < .04  < .59 | 0.02; 0.91  -0.01; 0.01 |
| **Eco-anxiety scale**  Intercept (Reference: CAS)  HEAS  Test of moderators  Heterogeneity | 24  *F*(1, 22) = 3.77, *p* = 0.07. *R^2^* = 13.85%  *Q* = 163.24, *p* < .0001. . τ2 = 0.02. *I^2^* = 86.50% | 0.34  0.13 | 0.04  0.07 | 9.12  1.94 | < .0001  < .07 | 0.26; 0.42  -0.01; 0.27 |
| **Economic status**  Intercept (reference: High income)  Lower-middle  Upper-middle  Test of moderators  Heterogeneity | 25  04  04  *F*(2, 30) = 0.21, *p* = 0.81. *R^2^* = 0.00%  *Q* = 298.86 *p* < .0001. τ2 = 0.02. *I^2^* = 89.55% | 0.34  0.04  0.04 | 0.03  0.08  0.08 | 10.79  0.47  0.51 | < .0001  < .64  < .61 | 0.28; 0.41  -0.13; 0.21  -0.13; 0.21 |
| **Sex**  Intercept  Female %  Test of moderators  Heterogeneity | 29  *F*(1, 27) = 0.04, *p* = 0.85. *R^2^* = 0.00%  *Q* = 310.96, *p* < .0001. τ2 = 0.02. *I^2^* = 90.02% | 0.40  -0.00 | 0.20  0.00 | 1.99  -0.20 | < .06  < .85 | -0.01; 0.81  -0.01; 0.01 |
| **Mean age**  Intercept  Age  Test of moderators  Heterogeneity | 25  *F*(1, 23) = 0.55, *p* = 0.46. *R^2^* = 0.00%  *Q* = 245.69, *p* < .0001. τ2 = 0.01. *I^2^* = 89.85% | 0.20  0.01 | 0.25  0.01 | 0.81  0.74 | < .43  < .46 | -0.31; 0.71  -0.01; 0.03 |
| **University education**  Intercept  University education %  Test of moderators  Heterogeneity | 20  *F*(1, 18) = 0.01, *p* = 0.94. *R^2^* = 0.00%  *Q* = 261.24, *p* < .0001. τ2 = 0.03. *I^2^* = 91.61% | 0.36  0.00 | 0.17  0.00 | 2.13  0.08 | < .05  < .94 | 0.00; 0.72  -0.00; 0.00 |
| **Risk of bias**  Intercept (reference: High risk of bias)  Low risk of bias  Test of moderators  Heterogeneity | 33  *F*(1, 31) = 0.14, *p* = 0.71. *R^2^* = 0.00%  *Q* = 272.78, *p* < .0001. τ2 = 0.02. *I^2^* = 89.26% | 0.32  0.03 | 0.08  0.08 | 4.29  0.38 | < .00  < .71 | 0.17; 0.48  -0.13; 0.20 |

**Table A.7.3.** Meta-regression analyses for Eco-anxiety and Stress. Note: k: Number of studies. SE: Standard error. p: Significance value. CI: Confidence intervals. R^2^: Proportion of variance explained.

| **Moderators** | ***k*** | **Estimate** | **SE** | **t** | ***p*** | **95% CI** |
| --- | --- | --- | --- | --- | --- | --- |
| **Population**  Intercept (Reference: general population )  University students  Test of moderators  Heterogeneity | 10  *F*(1, 8) = 0.01, *p* = 0.91. *R^2^* = 0.00%  *Q* = 141.83, *p* < .0001. τ2 = 0.02. *I^2^* = 92.38% | 0.32  0.01 | 0.08  0.10 | 4.08  0.11 | < .00  < .91 | 0.14; 0.50  -0.22; 0.24 |
| **Climate risk vulnerability**  Intercept  Climate risk vulnerability  Test of moderators  Heterogeneity | 10  *F*(1, 8) = 0.00, *p* = 0.97. *R^2^* = 0.00%  *Q* = 136.71, *p* < .0001. τ2 = 0.02. *I^2^* = 92.29% | 0.35  -0.07 | 0.62  1.96 | 0.56  -0.03 | < .59  < .97 | -1.07; 1.76  -4.59; 4.46 |
| **Climate risk readiness**  Intercept  Climate risk readiness  Test of moderators  Heterogeneity | 10  *F*(1, 8) = 0.66, *p* = 0.44. *R^2^* = 0.00%  *Q* = 118.59, *p* < .0001. τ2 = 0.02. *I^2^* = 91.68% | -0.04  0.57 | 0.45  0.71 | -0.09  0.81 | < .93  < .44 | -1.08; 1.00  -1.05; 2.20 |
| **Climate risk ND-GAIN Index**  Intercept  Climate risk ND-GAIN Index  Test of moderators  Heterogeneity | 10  *F*(1,8) = 0.39, *p* = 0.55. *R^2^* = 0.00%  *Q* = 122.23, *p* < .0001. τ2 = 0.02. *I^2^* = 91.88% | -0.13  0.01 | 0.73  0.01 | -0.18  0.63 | < .86  < .55 | -1.82; 1.55  -0.02; 0.03 |
| **Sex**  Intercept  Female %  Test of moderators  Heterogeneity | 9  *F*(1, 7) = 1.36, *p* = 0.28. *R^2^* = 5.39%  *Q* = 87.91, *p* < .0001. τ2 = 0.02. *I^2^* = 91.26% | -0.13  0.01 | 0.38  0.01 | -0.34  1.17 | < .75  < .28 | -1.02; 0.77  -0.01; 0.02 |
| **Mean age**  Intercept  Age  Test of moderators  Heterogeneity | 8  *F*(1,6) = 0.02, *p* = 0.90. *R^2^* = 0.00%  *Q* = 138.39, *p* < .0001. τ2 = 0.03. *I^2^* = 94.43% | 0.25  0.00 | 0.57  0.03 | 0.43  0.13 | < .68  < .90 | -1.14; 1.63  -0.06; 0.07 |

**Appendix A8**

Funnel plots of meta-analytic estimates:

**Figure A.8.1.** The contour-enhanced funnel plots for meta-analysis of the relationship between eco-anxiety and depression

**Figure A.8.2.** The contour-enhanced funnel plots for meta-analysis of the relationship between eco-anxiety and anxiety

**Figure A.8.3.** The contour-enhanced funnel plots for meta-analysis of the relationship between eco-anxiety and stress

**Figure A.8.4** The contour-enhanced funnel plot with trim and fill method for eco-anxiety and depression


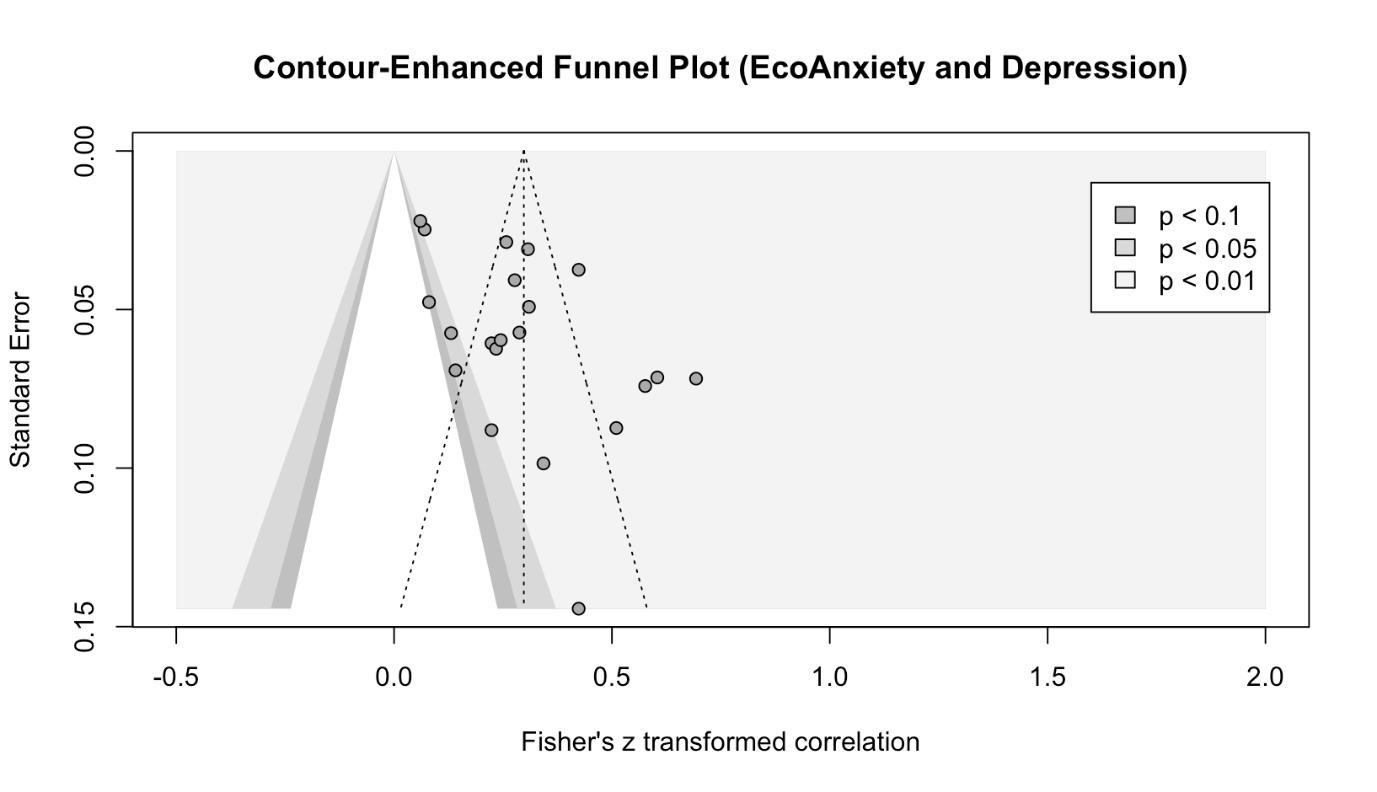


**Figure A.8.1.** The contour-enhanced funnel plots for meta-analysis of the relationship between eco-anxiety and depression


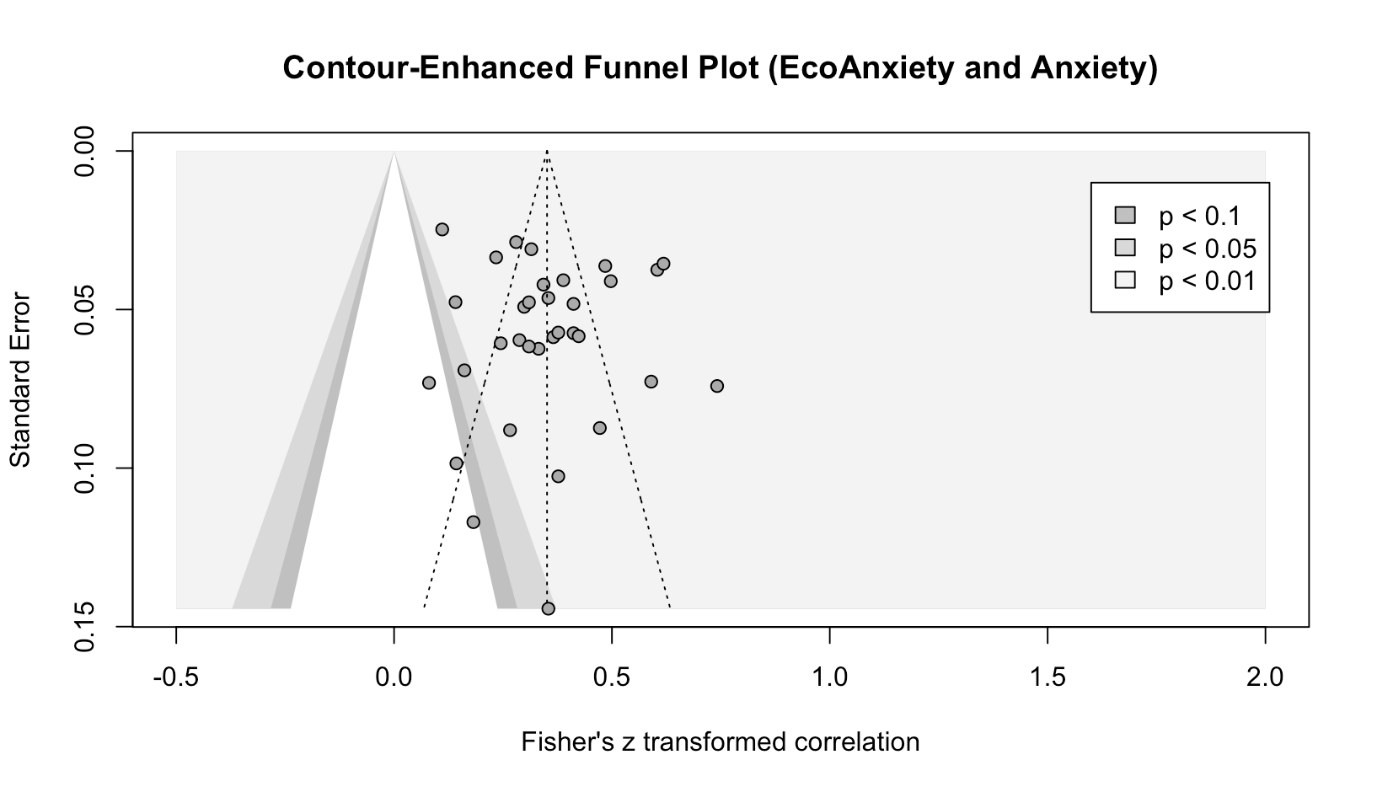


**Figure A.8.2.** The contour-enhanced funnel plots for meta-analysis of the relationship between eco-anxiety and anxiety


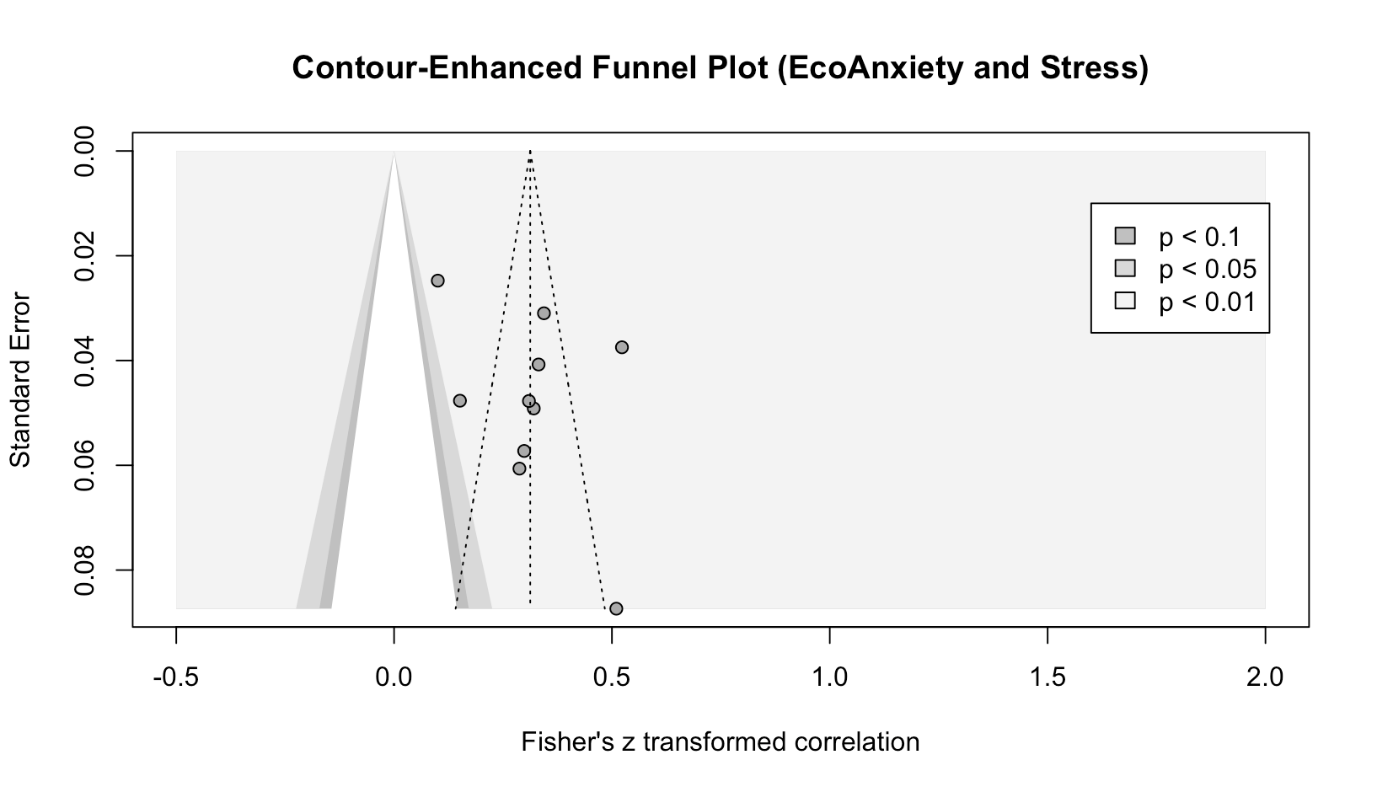


**Figure A.8.3.** The contour-enhanced funnel plots for meta-analysis of the relationship between eco-anxiety and stress


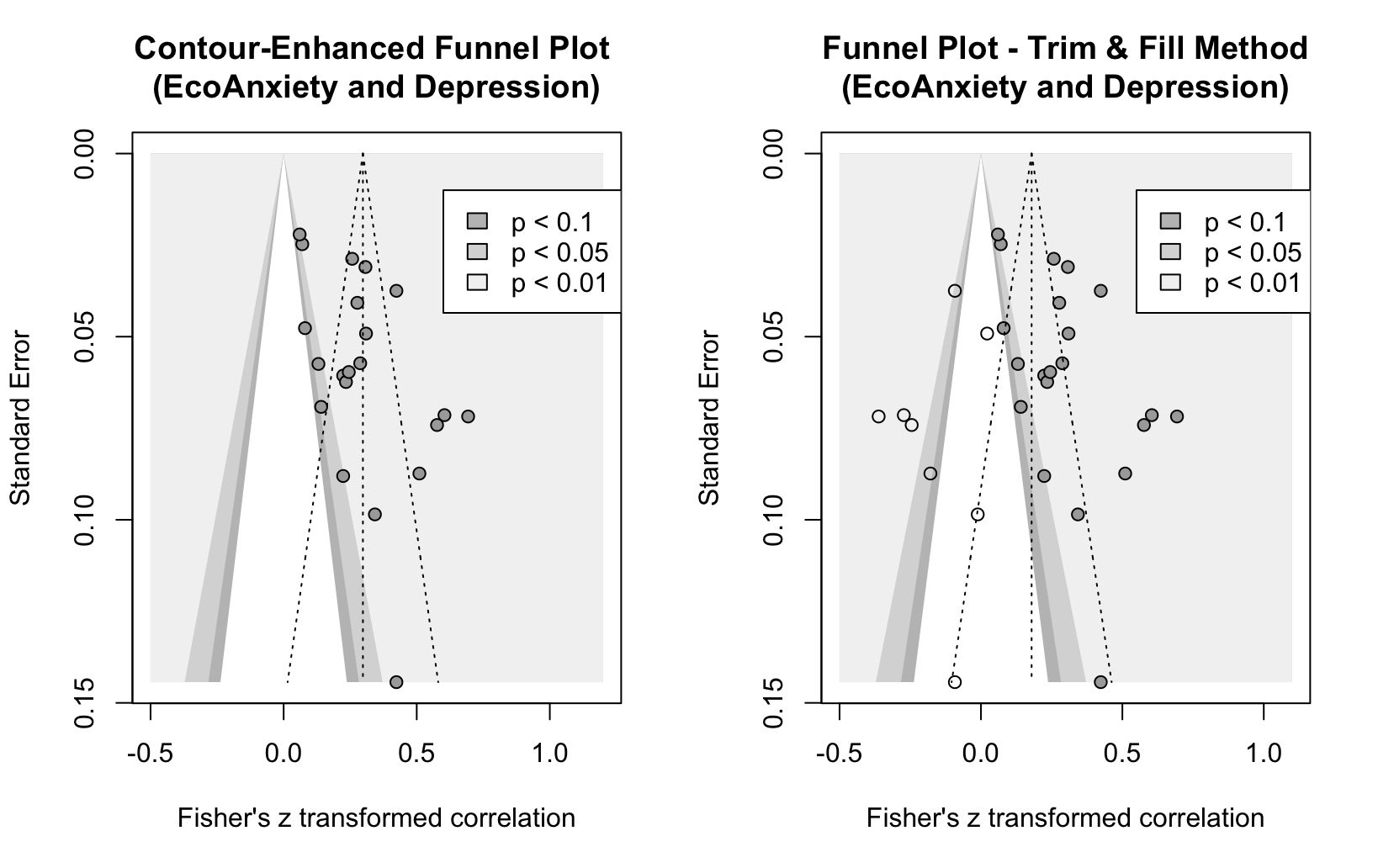


**Figure A.8.4.** The contour-enhanced funnel plot with trim and fill method for eco-anxiety and depression
